# The Effect of Sr, Ti, and B on the Crystallization Process and Mechanical Properties of the AlSi9Cu3(Fe) Alloy

**DOI:** 10.3390/ma18040882

**Published:** 2025-02-17

**Authors:** Tomasz Szymczak, Bogusław Pisarek, Cezary Rapiejko, Ryszard Władysiak, Paweł Just, Rafał Kaczorowski, Grzegorz Gumienny, Bartłomiej Januszewicz, Jarosław Piątkowski, Viktor Sinelnikov, Tadeusz Pacyniak

**Affiliations:** 1 Department of Materials Engineering and Production Systems, Lodz University of Technology, 90-924 Lodz, Poland; cezary.rapiejko@p.lodz.pl (C.R.); ryszard.wladysiak@p.lodz.pl (R.W.); pawel.just@p.lodz.pl (P.J.); rafal.kaczorowski@p.lodz.pl (R.K.); grzegorz.gumienny@p.lodz.pl (G.G.); tadeusz.pacyniak@p.lodz.pl (T.P.); 2 Institute of Materials Science and Engineering, Lodz University of Technology, 90-924 Lodz, Poland; bartlomiej.januszewicz@p.lodz.pl; 3 Department of Materials Technology, Silesian University of Technology, 40-019 Katowice, Poland; jaroslaw.piatkowski@polsl.pl; 4 Łukasiewicz Research Network—Institute of Ceramics and Building Materials, 31-983 Krakow, Poland; viktor.sinelnikov@icimb.lukasiewicz.gov.pl

**Keywords:** EN AC-46000 alloy, Sr and Ti modifiers, die casting, high-pressure die casting, crystallization, thermal and derivative analysis, mechanical properties, density index, optimization

## Abstract

This article presents studies on the effect of Sr and TiB on the crystallization process, mechanical properties, hardness, and density index of the Al-Si alloy from the EN AC-46000 group, with a narrowed chemical composition, produced by die-casting and HPDC (high-pressure die casting) technology. The research used the Box–Wilson method to design the experiment and stepwise multiple regression. To identify the optimal amount of Sr and Ti in the analyzed alloy that would simultaneously guarantee the maximization of UTS, YS, A_gt_, and HBW and the minimization of the DI (density index), multi-criteria optimization was performed. The modifiers were added to the liquid alloy as AlSr10 and AlTi5B1 master alloys. It was found that for 0.02–0.04 wt.% Sr and 0.05–0.08 wt.% Ti in the die castings, the highest mechanical properties, such as UTS, YS, A_gt_, and HBW (treated as stimulants in the experiment), can be obtained simultaneously with the lowest alloy gasification identified by DI (treated as a destimulant in the experiment). It was also confirmed that the same amount of the above-mentioned elements in HPDC castings caused an increase in UTS by approx. 14%, YS by approx. 6%, A by approx. 47%, and HBW by approx. 13%, with a relatively small increase in DI by approx. 5% compared to the unmodified alloy.

## 1. Introduction

In Al-Si alloys, strontium, titanium, and boron are used to modify and refine the phases in the microstructure. The modification aims to create favorable conditions for microstructure refinement and/or favorable phase morphology, mainly silicon. The modification of hypoeutectic and eutectic Al-Si alloys consists of changing the morphology of the eutectic silicon from lamellar to fibrous or reducing the interphase distance λ of the α_Al_ + β(Si) eutectic mixture [1,2,3,4,5,6,7].

The morphology of eutectic silicon in the form of thin fibers is obtained by using sodium or strontium [1,2,3,4,5,6,7,8,9,10]. The advantage of strontium over sodium is its long-term action. Its ability to modify Si morphology persists even after multiple remelting cycles. The most used modifier reducing the interphase distance of the α_Al_ + β(Si) eutectic mixture is antimony. The modification of both Sr and Sb significantly increases the tensile strength and elongation and slightly increases the hardness of Al-Si alloy [2,6].

Additionally, alloys whose microstructure contains dendrites of the α_Al_ solid solution can be modified with titanium and boron. Both titanium [8,9] and boron [11] can modify α_Al_ dendrites. However, the greatest refinement is achieved when these elements are used simultaneously. The appropriate addition of Ti and B causes an effective reduction in the size of α_Al_ dendrites [6,8,9,11].

**Figure 1:**
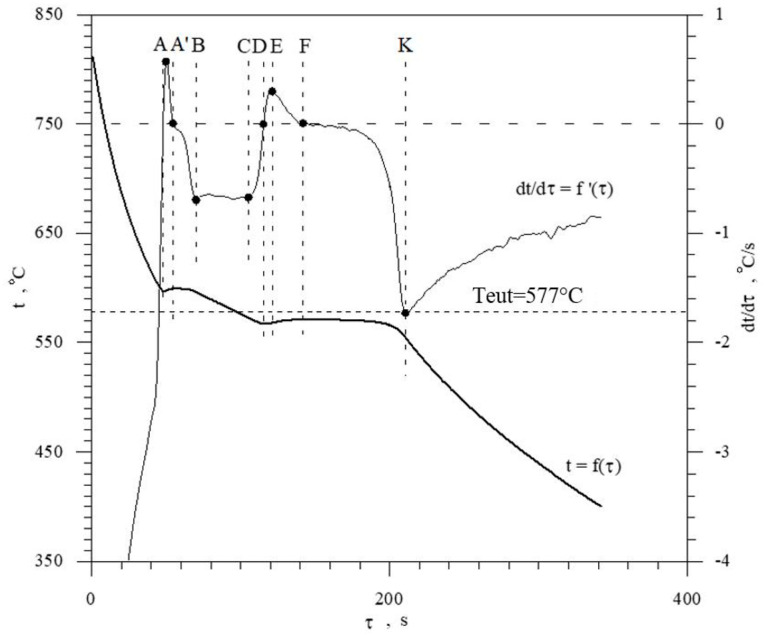
Representative TDA curves of the hypoeutectic AlSi7 alloy.

The discussed elements that modify and refine the phases of Al-Si alloys significantly affect their crystallization process. Figure 1 shows the crystallization process of an exemplary unmodified hypoeutectic AlSi7 alloy recorded by thermal and derivative analysis (TDA).

The figure shows two curves: t = f(τ) temperature with respect to time and its derivative dt/dτ = f′(τ) with marked characteristic points. These points are mainly local extrema of the derivative “dt/dτ” and the so-called zeros dt/dτ = 0. Point A determines the maximum thermal effect caused by the crystallization of the solid solution α_Al_, i.e., the moment of the most intense release of the latent heat of crystallization. Between points B and C, the crystallization of the α_Al_ phase stabilizes, the temperature of the solid–liquid mixture decreases approximately uniformly, and the kinetics of thermal crystallization processes dt/dτ ≈ const. Then, the liquid is supercooled relative to the equilibrium eutectic transformation temperature T_eut_ = 577 °C (for the Al-Si system), which initiates the onset of the α_Al_ + β(Si) binary eutectic crystallization at point C. Points D and F are the temperature recalescence boundaries at t = f(τ) during the crystallization of the α_Al_ + β(Si) binary eutectic. These are the points occurring when the minimum (point D) and maximum (point F) temperatures are recorded during the crystallization of the α_Al_ + β(Si) eutectic mixture. The temperature difference between points F and D indicates temperature recalescence. Point E determines the maximum thermal effect of crystallization of the eutectic mixture, while point K determines the end of the binary eutectic crystallization and in this case the end of the liquid alloy solidification in the volume of the TDA probe.

**Figure 2:**
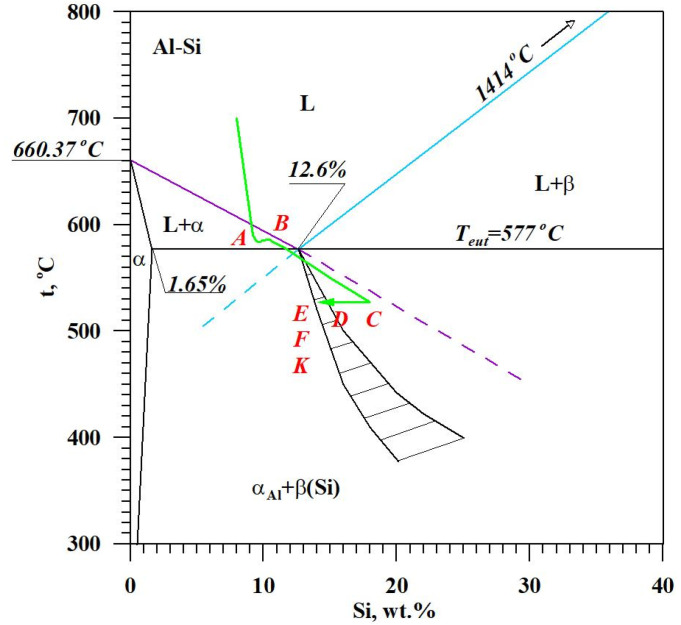
The crystallization scheme of the hypoeutectic Al-Si alloy and the region of the coupled growth of the α_Al_ + β(Si) binary eutectic mixture.

The diagram of the hypoeutectic alloy crystallization and the region of coupled growth of the α_Al_ + β(Si) eutectic mixture are shown in Figure 2. The crystallization process of the hypoeutectic Al-Si alloy starts with the nucleation and growth of the α_Al_ solid solution dendrites from a liquid overcooled vs. liquidus line. The heat of α_Al_-phase crystallization causes temperature recalescence, which terminates at point A′ (Figure 1). The crystallization front of α_Al_ dendrites “rejects” Si atoms into the liquid, causing it to become richer in this element. After exceeding the above-mentioned point, the temperature of the liquid decreases, which leads to the initiation of α_Al_ + β(Si) eutectic mixture crystallization. The leading phase during this crystallization is silicon, which is a good catalyst for the nucleation of the α_Al_ phase. Point D represents the entrance into the region of the coupled growth of the α_Al_ + β(Si) eutectic mixture. The crystallization heat of the binary eutectic mixture causes its maximum thermal effect to take place at point E, and the temperature rises to a value at point F. Crystallization terminates at point K.

This article will present a comparison of the crystallization course of an exemplary unmodified hypoeutectic alloy and those modified with different Sr, Ti, and B contents.

The published research results on the crystallization process of hypo- and eutectic Al-Si alloys show that strontium decreases the crystallization temperature of the αAl+β(Si) eutectic mixture as well as affects the change of temperature recalescence in the eutectic region [5,6,12]. Thermal and derivative analysis (TDA) was used to study the crystallization process in the above-mentioned papers. For example, in [12], it was reported that the addition of 0.04% Sr to the AlSi7Mg0.3Fe0.8 alloy caused a decrease in the crystallization temperature of the α_Al_ + β(Si) eutectic mixture by 8.7 °C (this effect was described as eutectic Si). Information regarding the effect of Sr on the recalescence of the eutectic crystallization temperature is divergent. The authors of [5] state that Sr increases the recalescence temperature of the α_Al_ + β(Si) eutectic mixture, while the results presented in [6] indicate its reduction. According to the data from [6], the simultaneous addition of Ti and B into the hypoeutectic alloy reduces the thermal effect of α_Al_ solid solution crystallization. In the same paper, it was also reported that the simultaneous addition of Sr, Ti, and B increases the temperature recalescence during the crystallization of the α_Al_ + β(Si) eutectic mixture and increases the thermal effect of the αAl phase crystallization. Studies on hypoeutectic and eutectic Al-Si alloys containing 0.03–0.37% titanium can be found in [9,13,14,15,16,17], while the papers [5,6,7,12,13,14,17] contain studies on strontium in amounts from 0.001% to 0.30%. These results apply to hypoeutectic alloys of the AlSi7Mg, AlSi7Mg0.3Cu0.5 and AlSi10 grades, as well as to the eutectic AlSi12Fe grade. The presented results show that the use of the above-mentioned elements separately, together, and with other modifying or grain-refining elements; e.g., B [11,12,14] or Zr [15,16,17] has a positive effect on mechanical properties such as: UTS, YS, A_gt_, HB, or fatigue strength. However, previous studies in this area have not used multi-criteria analysis to evaluate the effectiveness of Sr and TiB modification of Al-Si alloy in terms of simultaneous maximization of several selected mechanical and quality properties.

Therefore, the aim of this paper is to investigate the effect of Sr and TiB modifiers on the crystallization process of the AlSi9Cu3(Fe) alloy (EN AC-46000) using thermal and derivative analysis and to optimize the amount of modifiers vs. the simultaneous maximization of its mechanical (UTS, YS, A_gt_, HB) and qualitative (DI—density index) properties. Statistical methods with multi-criteria analysis were used to achieve this goal.

## 2. Materials and Methods

**Figure 3:**
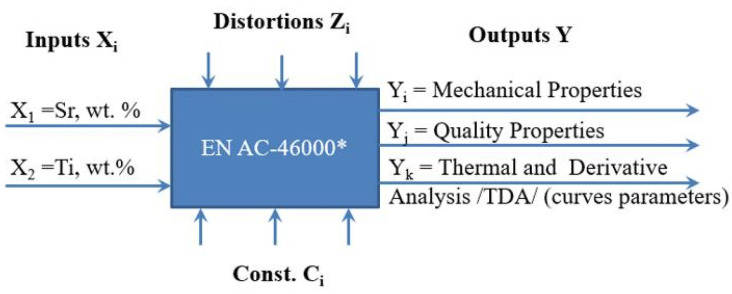
General experiment model representing the test object including inputs X_i_, outputs Y_i_, process parameter constants Ci, and interference Z_i_.

**Figure 4:**
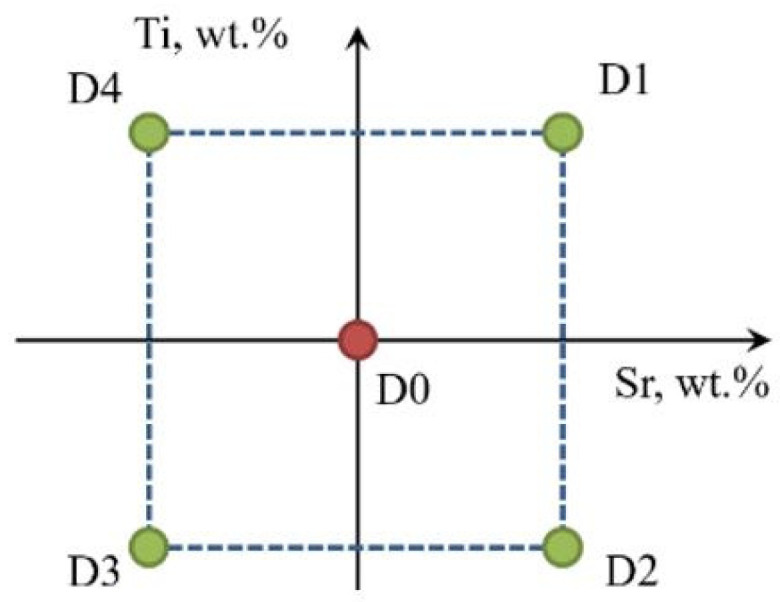
Graphical representation of the experimental plan in the Ti-Sr concentration system.

The general experimental model is presented in Figure 3, while the experimental plan is presented in Figure 4 and Table 1.

There is little understood the effect of high Sr concentration in Al-Si alloys cast by gravity die casting technology and especially by high-pressure die casting (HPDC) technology. The use of the Box–Wilson method to determine the gradient vector of the most intense changes in the response of the object (UTS, YS, A_gt_, HBW, DI) to a given input (Si wt.%, Ti wt.%) was guided by setting values (Si wt.%, Ti wt.%) for the central task to cover the entire known range of Sr concentrations introduced into the Al-Si alloy as a modifier at the +Δ and -Δ levels. The paper [18] investigated the effect of Sr addition at 50–3000 ppm (0.005–0.3%) on the nucleation of eutectic mixture in high-purity Al-Si alloys. For this reason, we decided to adopt for the central task a concentration of Sr = 0.20 wt.% and ΔSr = 0.05 wt.% to approach 0.3% at the +Δ level, and at the −Δ level to approach the most used range of Sr concentrations in Al-Si alloys. Only at the optimization stage was the plan to analyze the range of 0–0.5 wt.% or 0.25–0.30 wt.% depending on the optimization results.

In Al-Si alloys, the effect of Ti on their microstructure and wear resistance was studied up to about 4 wt.% Ti [19]. With this amount, titanium forms intermetallic phases. Only a smaller addition of Ti at levels up to about 1.5 wt.% has a modifying effect on the microstructure of Al-Si alloys (an alloy that solidifies in an electric field) [20]. Based on studies [6,8,9] related to the effect of Ti as a modifier in Al-Si alloys, the same range of Ti concentration in the studied alloy was assumed as for Sr.

The EN AC-46000* grade alloy was tested with a narrowed content of Si, Cu, and Mg compared to the EN AC-46000 alloy specified in UNE EN 1706:2020 standard [21] —a typical Al-Si alloy for high-pressure die casting (HPDC). Due to the improvement in the mechanical properties of the tested alloy demonstrated in preliminary tests, the contents of its basic constituent elements were limited to the ranges: Si = 9.0–10.0 wt.%; Cu = 2.0–2.5 wt.% and Mg = 0.3–0.4 wt.%. The chemical composition range of the tested EN AC-46000* basic alloy is presented in Table 2.

The object in the general model of the experiment (Figure 3) is an EN AC-46000 alloy. It is acted upon by a variable input in the form of variable amounts (wt.%) of Sr and Ti modifiers selected in such a way as not to exceed their planned concentration in the alloy. The mass concentration of Sr and Ti in the alloy obtained after the addition of modifiers in the form of master alloys was assumed as the input factors X_i_. However, it is important to keep in mind that titanium was added as the AlTi5B1 master alloy, so it resulted in the simultaneous addition of boron at an amount of 1/5 of titanium. The nominal Sr and Ti contents in the individual melts (Table 1) were determined based on a two-level experimental design according to the Box–Wilson method. The aim of the experiment was to optimize Sr and Ti content. Based on the experiment conducted, the inputs significantly affect the outputs of the object, which are, respectively:

- Mechanical properties—UTS, YS, A_gt_ and HBW,
- Qualitative properties—DI (density index),
- TDA characteristic parameters.

The optimization criterion was to obtain the highest possible mechanical properties (UTS, YS, A, HBW). The following initial properties were adopted for the analysis: Y_i_ mechanical—tensile strength (UTS); yield strength (YS); relative elongation (A_gt_) and hardness (HBW), Y_j_ qualitative—density index DI; and Yk—characteristic quantities determined from TDA characteristics: t = f(τ) and dt/dτ = f′(τ). The following were taken as characteristic quantities of TDA curves: alloy temperature “t”, time from the beginning of the measurement “τ”, the first derivative “K = dt/dτ” of temperature “t” with respect to time “τ”, and “Z = d_2_t/dτ^2^” the second derivative of temperature “t” with respect to time “τ” describing the thermal processes of the tested alloy solidifying and cooling in the TDA probe.

The optimization of the alloy modification was performed in two steps. In the first one, based on a two-level experimental design, the response of the object in the vicinity of the central point D0 at the levels “+ΔX_i_” and “−ΔX_i_” generating the maximum response Y_i_ and the minimum response Y_j_ was sought. For this purpose, the Equations of the planes (1) containing the gradient vectors of the most intensive changes in the object’s $\vec{{\nabla Y}_{i}}i\nabla \vec{Y_{J}}$ response to the given inputs X_i_ were determined using the multiple regression analysis method.

(1) $$Y_{i\;or\;j}=a_{0}+a_{1}\cdot X_{1}+a_{2}\cdot X_{2}$$

For the gradient vectors $\vec{{\nabla Y}_{i}}i\nabla \vec{Y_{J}}\;$ determined based on Equation (1) for individual outputs, the resultant vector $\vec{\nabla GR}$ was determined, and, according to the Equation of the straight line (2) on which it lies, the input values for additional tasks (D5 and D6) were estimated.

(2) $$Ti=a_{0}+a_{1}\cdot Sr$$

In the first stage of optimization, the criterion of the simultaneous maximization of the outputs Y_i_ (UTS, YS, A_gt_, HBW) and minimization of the output Y_j_ (DI) was adopted.

In the second stage of optimization, based on tasks lying along the designated vector $\vec{\nabla GR}$: D0, D5, and D6 (with settings changing in line with the direction of the gradient vector) and tasks U and Dx (located closest to the designated direction), a mathematical description was sought as a function with one extremum—a parabola (3):

(3) $$Y_{i\;or\;j}=a_{0}+a_{1}\cdot X_{1}+a_{2}\cdot X_{2}+a_{3}\cdot X_{1}\cdot X_{2}$$

The coordinates of the parabola vertex (X1, X2) take on the optimal values X_i_ for alloy modification as a function of the selected response $Y_{i\;or\;j}$. For this reason, the obtained response models (3) (UTS, YS, A_gt_, HBW, DI) were tabulated for selected ranges of input factor variability (Ti and Sr). Since the condition of obtaining simultaneously the highest possible mechanical properties (Y_i_) with the highest possible alloy quality (the lowest possible DI coefficient) was taken as the optimality criterion, the multi-criteria optimization method was used. The normalization of Y_i_ and Yj responses was carried out by determining for Y_i_ the stimulant values nY_i_ while for Y_j_ the destimulant value nY_j_ according to Formulas (4) and (5):

(4) $${nY}_{i}=\frac{Y_{i}-Y_{i}\_min}{Y_{i}\_max-Y_{i}\_min}$$

(5) $${nY}_{j}=\frac{Y_{j\_max}-Y_{j}}{Y_{j}\_max-Y_{j}\_min}$$

For normalized response values $Y_{i\;or\;j}$ for selected ranges of input factor variability (Ti and Sr), an additional column was added with the value of the objective function.

For the Fo objective function, the condition of its maximization was assumed according to Formula (6):

(6) $$Fo=\sum_{i}{nY}_{i}+\sum_{j}{nY}_{j}\to max$$

$$i\in \left\{1,2,3,4\right\}\;and\;j=1.$$

The values of the input factors (Ti, wt.%, Sr, wt.%) for which the largest value of the Fo_max_ objective function was obtained represent the optimal values (Ti, Sr)_opt_ for the adopted optimality criterion (6).

Melting parameters were taken as Const. C_i_ parameters (Figure 3). The base alloy ingots were smelted in a PI30 Elkon induction furnace (Ekon, Rybnik, Poland) in an AC20 crucible made of SiC. The volume of the crucible was about 7 kg of Al alloy. After smelting, the alloy was superheated to 700 ± 10 °C; then, Sr and Ti and B were added as AlSr10 (Sr = 9.7 wt.%; Fe = 0.28 wt.% and Si = 0.05 wt.%) and AlTi5B1 (Ti = 4.95 wt.%; B = 0.95 wt.%; Si = 0.19 wt.% and Fe = 0.14 wt.%) master alloys, respectively.

The object is affected by an unmeasurable disturbance Z_i_, for which it is not possible to determine the probability of occurrence. For example, the voltage fluctuations in the electrical network supplying the induction coil of the furnace melting the metal charge of the alloy being tested affect the process of melting the charge and the intensity of its mixing, and a change in the speed of air movement in the vicinity of the TDA probe affects the process of cooling the alloy.

The selection of predictors (parameters determined on the characteristics t = f(τ) was dt/dτ = f′(τ) for points describing as a function of time τX; temperature tX; kinetics KX and dynamics ZX of thermal processes of the alloy solidifying and cooling in the probe TDA/X = {A, B, C,...}/) of regressions describing the change of UTS, YS, A_gt_, HBW, and DI as a function based on the procedure of the Regression Model Selection. In Statgraphics, “the Regression Model Selection procedure of statistical data mining fits models involving all possible linear combinations of a set of predictors all selects the best models using criteria such as Mallows’ Cp and the adjusted R-squared statistic” [22]. Only those members (predictors) for which the adjusted R-squared statistic reached the highest value were entered into the regression Equations. Then, if necessary (with a lack of significance of the regression function or any member of the regression function), two Key Selection Procedures were used: [22]:

- “Forward Selection—starts with no variables and adds them one by one, beginning with the one most correlated with the outcome. Variables considered more important are added first and remain in the model”.
- “Backward Selection—begins with all variables in the model, removing them one at a time if they don’t enhance the regression Equation”.

Validation (evaluation) of the models was based, for the adopted level of statistical significance α, on the evaluation of the significance of the regression function F_model_ > F(α, ν_1_, ν_2_); the evaluation of the significance of the individual members a_i_/i = {0,1,2,3,...—of the regression function P__value ai_ ≤ α; the evaluation of the variability of the data—square of the correlation coefficient R^2^—the closer to 1 the better; standard error of estimation SEE—the smaller the better; mean total error MAE—the smaller the better.

An analysis of the object’s response to a change in input quantities indicates a search for optimal values of mechanical properties in the direction of decreasing Ti and Sr contents outside the experimental plan. Therefore, two additional optimization melts were made for lower contents of these elements than assumed in the experimental plan. These were D5 and D6 tasks with nominal Ti and Sr contents of 0.10 wt.% and 0.05 wt.% each, respectively. The actual chemical composition for each melt, confirmed by testing on the spark optical emission spectrometry (OES) analyzer, is shown in Table 3. As a reference point for analyzing the test results, the base alloy smelting without the addition of Sr, Ti, and B, designated as U (Unmodified) in Table 3, was also performed. In runs, D0-D4, the chemical composition of the melts from the experiment plan is presented (Table 1, gradient vector determination). Runs D5 and D6, highlighted in bold and italic font, because they show the chemical composition of the melts from the optimization stage of the modification of the studied alloy.

**Figure 5:**
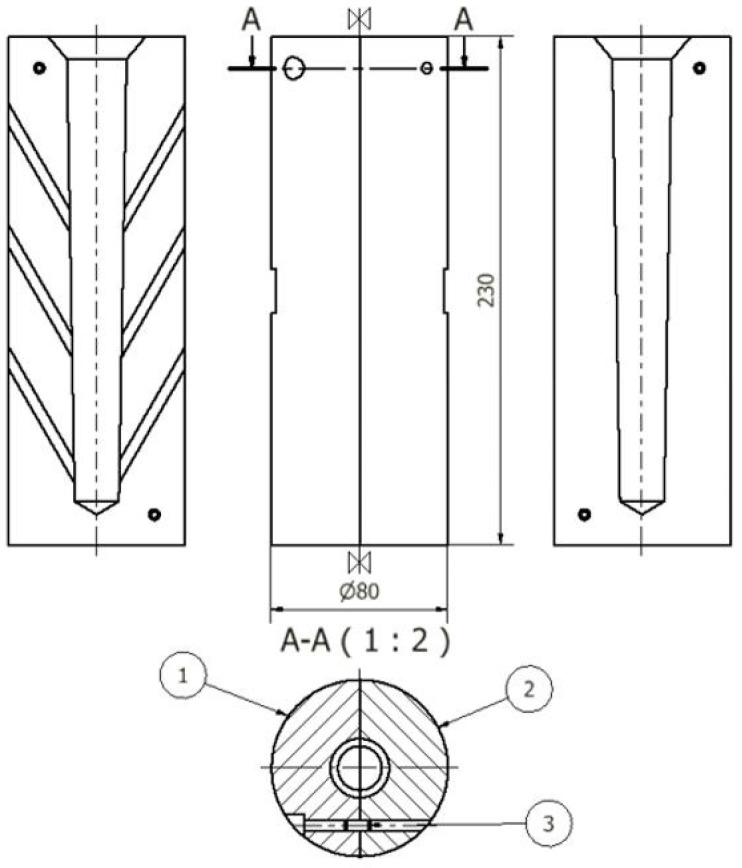
Die for test castings Ø20: 1—left part, 2—right part, 3—dowel pin (units: mm).

**Figure 6:**
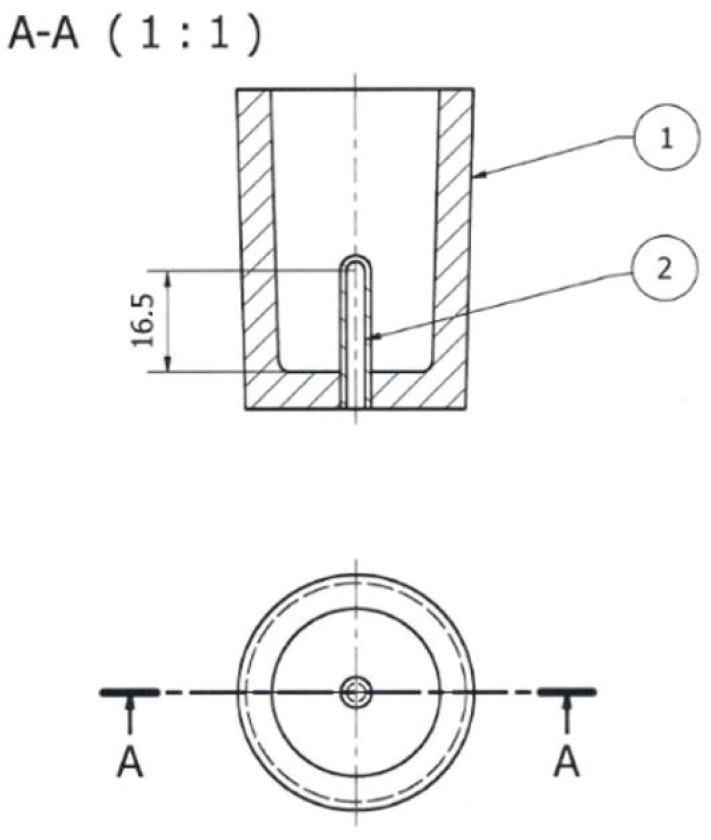
TDA probe: 1—shell mold; 2—quartz tube closed on one side (units: mm).

After the master alloys with Sr, Ti, and B dissolved, the alloy was refined using an Ecosal Al113.S solid refiner. This refiner was introduced at a rate of 0.5 wt.% by the weight of the charge. Then, to dehydrogenate the melt in the furnace crucible, nitrogen (N_2_) was purged using a steel lance for 10 min. After refining, test castings were made. The tested alloy was cast into a die (Figure 5) as well as a shell mold in the form of the TDA probe (Figure 6). The permanent mold had a cylindrical cavity with an average diameter of 20 mm and a height of 210 mm. Its temperature at the time of pouring was 180 °C. The shell mold was made of resin sand. The pouring temperature was 710 ± 10 °C.

**Figure 7:**
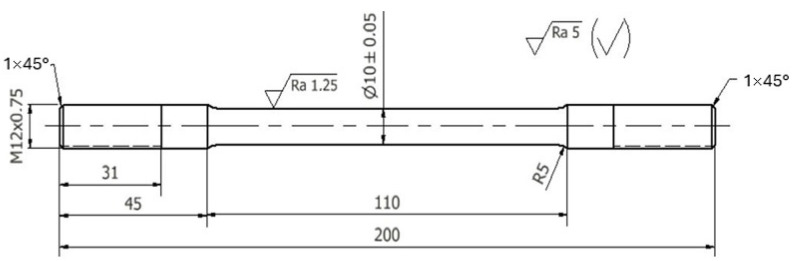
Dimensions of the strength test specimen.

Three die castings were made for each chemical composition tested. They were used to make static tensile test specimens. The machined specimens had shape and dimensions as shown in Figure 7.

The static tensile test was conducted in accordance with UNE EN ISO 6892-1:2020 [23] on an INSTRON 4485 testing machine. This test determined the tensile strength UTS, the yield strength YS, and the relative elongation A. The tests were conducted at a rate of stress increase of R = 6 MPa/s (permissible value of R = 2–20 MPa/s). The strain rate was 0.0008 m/s. Hardness was measured using the Brinell method in accordance with ISO 6506-1:2014 [24]. A tungsten carbide ball was used as an indenter. The measurement was carried out on a degreased flat and smooth surface, free of oxide layers and impurities. The loading force was selected so that the indentation diameter d was in the range of 0.24 D < d < 0.6 D. The diameter of the ball was 2.5 mm, the load was 613 N, and the static load holding time was 30 s.

**Figure 8:**
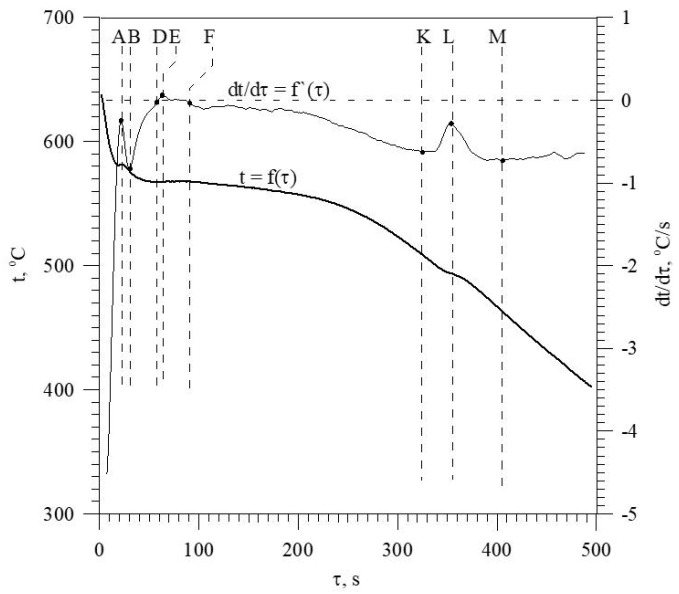
TDA curves of the EN AC-46000* base alloy.

Castings from the shell mold (Figure 6) were used for thermal and derivative analysis (TDA). This is a universal method used to study the crystallization process of alloys. PtRh10-Pt thermocouple (K-type) placed in a quartz tube in the thermal center of the TDA probe was used to record TDA curves. Figure 8 shows TDA curves of the unmodified Al-Si alloy (U—Table 3) EN AC-46000* refined with Ecosal Al113.S solid refiner and N_2_ gas refiner, with the characteristic points on the derivative curve dt/dτ = f′(τ) marked. These points are the local extrema of the derivative “dt/dτ” and the so-called zeros dt/dτ = 0. The characteristic points on the “dt/dτ” derivative curve are defined by time “τ” from the beginning of the measurement and temperature “t”. The interpretation of A, D, E and F points in this case (Figure 8) is analogous to the description of TDA curves in Figure 1. Point B represents the end of α_Al_ phase crystallization and, at the same time, the initiation of α_Al_ + β(Si) binary eutectic crystallization. The absence of A′ and C points (Figure 1) on TDA curves of EN AC-46000* base Al-Si alloy is due to the higher silicon content in the base alloy tested. The effect of increasing Si content is to reduce the thermal effect caused by the crystallization of the αAl solid solution and to reduce the distance between the thermal effects from this phase and the α_Al_ + β(Si) eutectic mixture. At the K point, there is the termination of eutectic mixture crystallization and the initiation of the crystallization of complex eutectic mixture containing intermetallic phases rich in Cu, Fe, and Mg. Point L represents the maximum thermal effect from the crystallization of this eutectic mixture, while point M defines the termination of this eutectic mixture crystallization and the end of the entire alloy solidification.

Metallographic specimens for microstructure examinations were prepared on specimens taken from castings made in the resin-coated sand probe and by high-pressure die casting. The surface of the specimens was etched with a 2% solution of hydrofluoric acid in water. The microstructure was examined using a Nikon Eclipse MA200 optical microscope (Nikon, Tokyo, Japan) with ×500 magnification for castings made in the resin-coated sand probe and by the die casting process, respectively.

Maps of the surface distribution of elements were made by EDS—energy-dispersive X-ray spectroscopy. For the study, a JEOL JSM-6610LV scanning microscope integrated with MiniCL-GATAN Cathodoluminescence Imaging System and Oxford Instruments EDS X-MAX 80 and NordlysMax electron backscatter diffraction (EBSD) was used. The research was conducted using AZtecEnergy’s EDS software ver. 4.4. The test specimens were placed in the microscope chamber, and the optimal test parameters were established, i.e., a working distance of 10 mm, an accelerating voltage of 20 kV, a beam current of 60, and an analysis time of 120 s.

To study the effect of Sr and Ti on the amount of gas in the alloy, the density index (DI) was determined. This test was conducted on Alu Speed Tester bench with FMA Balance (FMA Mechatronic Solutions AG, Schaan, Lichtenstein). The density index of the alloy was determined by measuring the density of specimens cast into the die on Alu Speed Tester bench. The first specimen solidified at ambient temperature and pressure, while the second solidified at reduced pressure to 80 mbar. The density of the specimens after cooling to ambient temperature, Datm for the first sample and D80mbar for the second sample, was measured on a dedicated FMA balance, and the DI index was automatically calculated using the Archimedes Principle.

From the determined densities of the specimens, the DI index can be determined from Formula (7):

(7) $$DI=\frac{D_{atm}-D_{80mbar}}{D_{atm}}\cdot 100,\%$$

where

*D_atm_*—density of the alloy specimen solidifying at the atmosphere, g/cm^3^;

*D_80mbar_*—density of the alloy specimens solidifying at reduced pressure up to 80 mbar, g/cm^3^.

## 3. Results and Discussion

The results presented in this section of the paper are a compilation of Y output parameter values in the adopted experimental model (Figure 3). These are the mechanical properties of the base and modified alloy: UTS, YS, A_gt_, and HBW; a quality parameter (DI (density index)); and TDA curves parameters. A general analysis of the effects of Sr and Ti modifiers on the microstructure of EN AC-46000* alloy under study is also presented.

### 3.1. Determination of Mechanical and Quality Parameters of the Alloy

The mechanical properties adopted for the analysis and density index were developed separately at both optimization stages.

#### 3.1.1. Al-Si Alloy Properties for the First Stage of Modifiers Content Optimization

**Figure 9:**
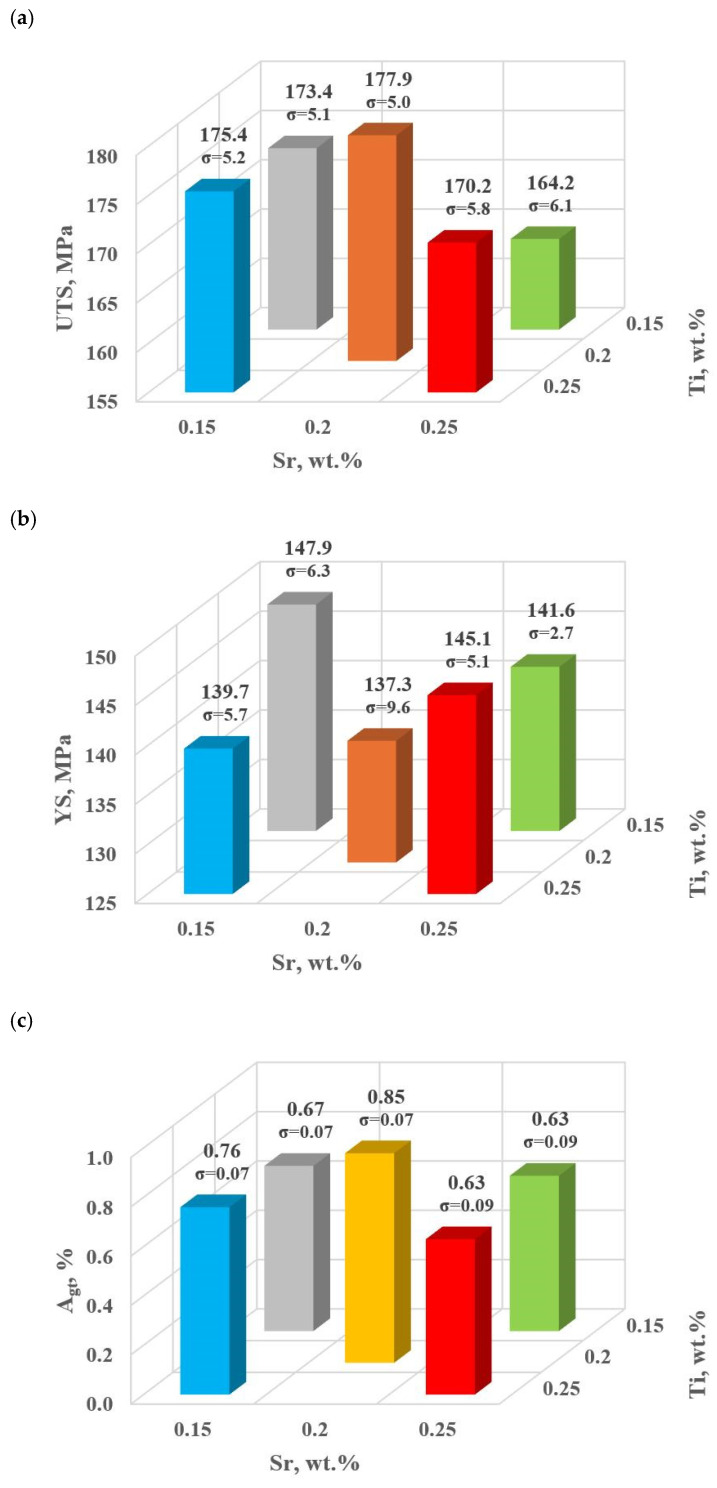

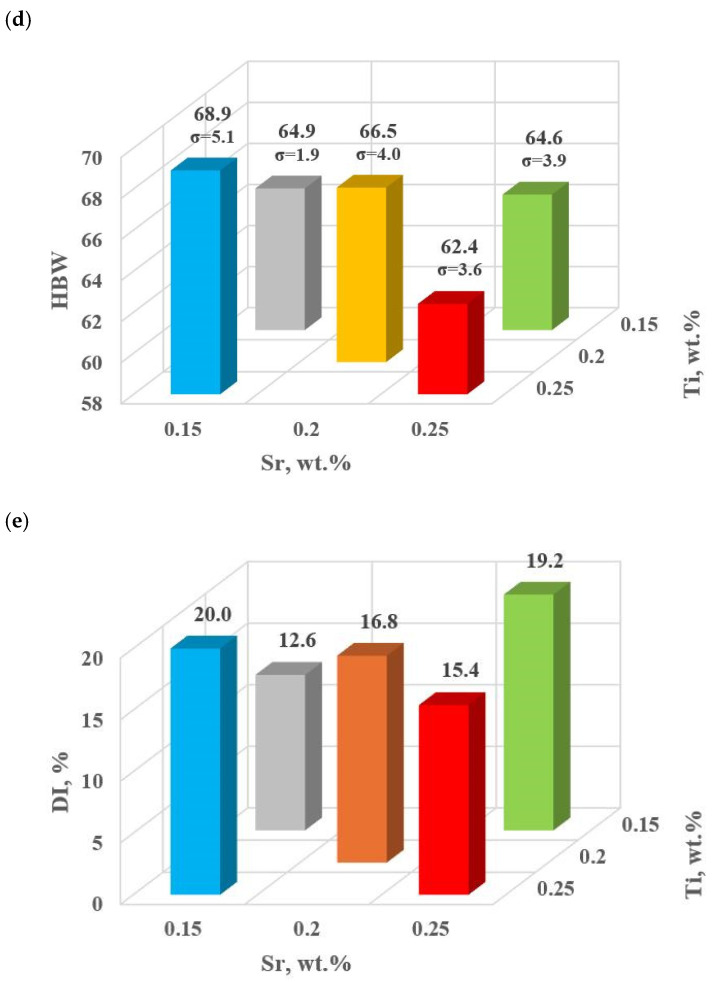
Basic mechanical properties of the tested EN AC-46000* alloy: (**a**) tensile strength UTS; (**b**) yield strength YS; (**c**) relative elongation A_gt_; (**d**) hardness HBW; (**e**) density index (DI).

This stage includes the experimental plan (tasks: D0–D4). Table 4 and Figure 9a–e show the mechanical and qualitative properties of the base alloy tested and with Sr and TiB added at the first optimization stage. Since with the addition of strontium, we expose it to a high degree of gassing [6], the DI (density index) was also included as a qualitative variable in the optimization analysis.

For the first stage of D0-D4 optimization, the extreme values for each mechanical and quality property have been color-coded to show that these extreme values are obtained at different modifier concentrations adopted in the D0-D4 tasks. The task of multi-criteria optimization—simultaneous maximization of UTS, YS, A_gt_, HBW, and minimization of DI—is to estimate such a concentration of modifiers that simultaneously achieves the highest possible mechanical properties with the lowest possible DI. However, these properties will not necessarily coincide in value with the determined local extremes (marked in Table 4).

From the data presented, the base alloy was characterized by tensile strength UTS = 187.0 MPa; yield strength YS = 150.0 MPa; relative elongation A_gt_ = 0.88% and hardness 72.0 HBW. The tested modifiers in amounts of 0.15–0.25 wt.% each (according to the experimental plan, these are runs: D0–D4) did not improve the tested properties of EN AC-46000* alloy. They also caused an increase in the amount of gas as evidenced by the lowest density index value of DI = 8.21 obtained for the unmodified alloy (U). The most favorable mechanical properties and density index obtained in the experiment (tasks: D0–D4) are marked in yellow in Table 4. From the data presented, the maximum values of UTS = 177.9 MPa and A_gt_ = 0.85% were obtained at the center point (task D0) for Sr and Ti contents of 0.20 wt.% each. The highest value of the contractual yield strength YS = 147.9 MPa and the lowest density index DI = 12.63% is characterized by the alloy from task D3, containing 0.15 wt.% Sr and Ti each. In contrast, task D4 (where Sr and Ti contents are 0.15 and 0.25 wt.%, respectively) yielded the highest hardness (HB = 68.9). Since the condition of obtaining simultaneously the highest mechanical properties and the lowest values of the DI (density index) was taken as the optimality criterion, to determine the direction of optimization of Sr and Ti content, the objective function Fo was calculated for each task according to Formula (4). The values of the Fo objective function for the tasks carried out in the first stage of optimization are shown in Table 5.

The highest value of Fo = 3.238 was obtained in task D3 (highlighted in bold in Table 5), that is, for minimal Sr and Ti content (0.15 wt. % each). Therefore, it should be inferred that the vector of the object’s most significant response to varying input quantity values is hooked in task D0 and directed toward decreasing Sr and Ti contents in task D3 (Figure 4). Considering the obtaining of both lower mechanical properties and a higher density index DI in the first stage of optimization (tasks D0–D4) compared to the unmodified alloy (U), it was decided to search for an optimal alloy outside the experimental plan established for the first stage of optimization. Therefore, to complete the optimization line for carrying out the second part of the optimization, two additional tasks D5 and D6 were determined, where Sr and Ti contents were 0.10 and 0.05 wt.% each, respectively.

**Figure 10:**
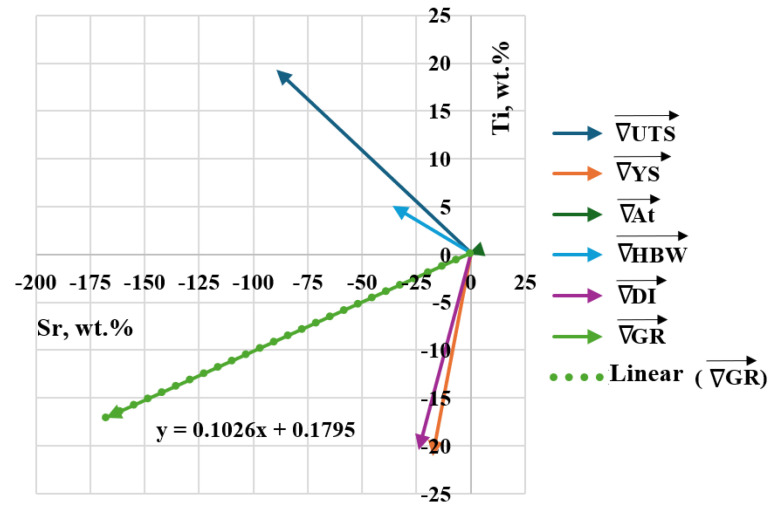
Gradient vectors Outputs ($\vec{{\nabla Y}_{i}}i\nabla \vec{Y_{J}}$) hooked to the point determined by task D0, the resultant gradient ($\vec{\nabla GR}$) and the mathematical model of y = f(x) line, where the resultant vector lies.

Table 6 shows the coordinates of the gradient vectors for each Output $\vec{{\nabla Y}_{i}}i\nabla \vec{Y_{J}}$ and the resultant gradient $\vec{\nabla GR}$ for the criterion of simultaneous maximization of stimulants (UTS, YS, A, HBW) and minimization of destimulant (DI). Graphically, the gradient vectors hooked to the point determined by D0 task are shown in Figure 10. The determined gradient vectors determine the direction of changes in the concentration of Sr and Ti modifiers allowing optimization of the selected (single) property of the alloy.

To determine the coordinates of tasks D5 and D6, the Formula (8) identified from the gradient vector $\vec{\nabla GR}\;$ was used:

(8) Ti=0.1795+0.1026·Sr

The theoretical setting values for tasks D5 and D6 are shown in Table 7.

**Figure 11:**
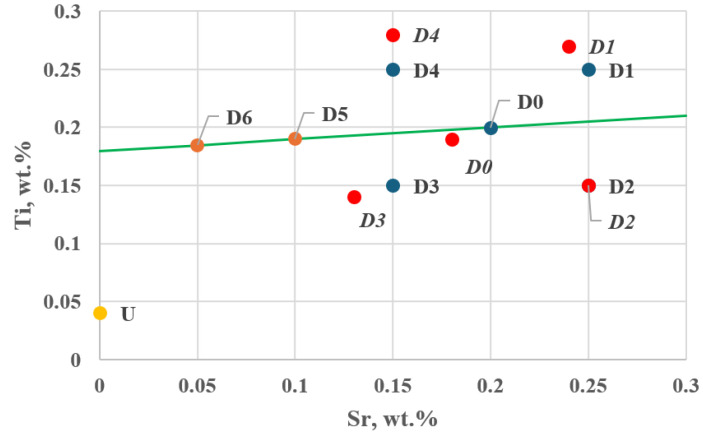
Position of additional tasks D5 and D6 along the direction and sense of the gradient vector $\vec{\nabla GR}$ in the experimental plan—Stage I (D0–D4—planned setting values, *D0*–*D4*—actual setting values), U—Ti and Sr concentrations in the unmodified alloy (wt.%).

Figure 11 shows the location of additional tasks in the experimental plan—Stage I.

Task D3 lies the closest to the direction determined by the resultant gradient vector $\vec{\nabla GR}$.

#### 3.1.2. Al-Si Alloy Properties for the Second Stage of the Modifiers Content Optimization

This stage includes additional melts (tasks: D5 and D6) to complete the optimization line.

**Figure 12:**
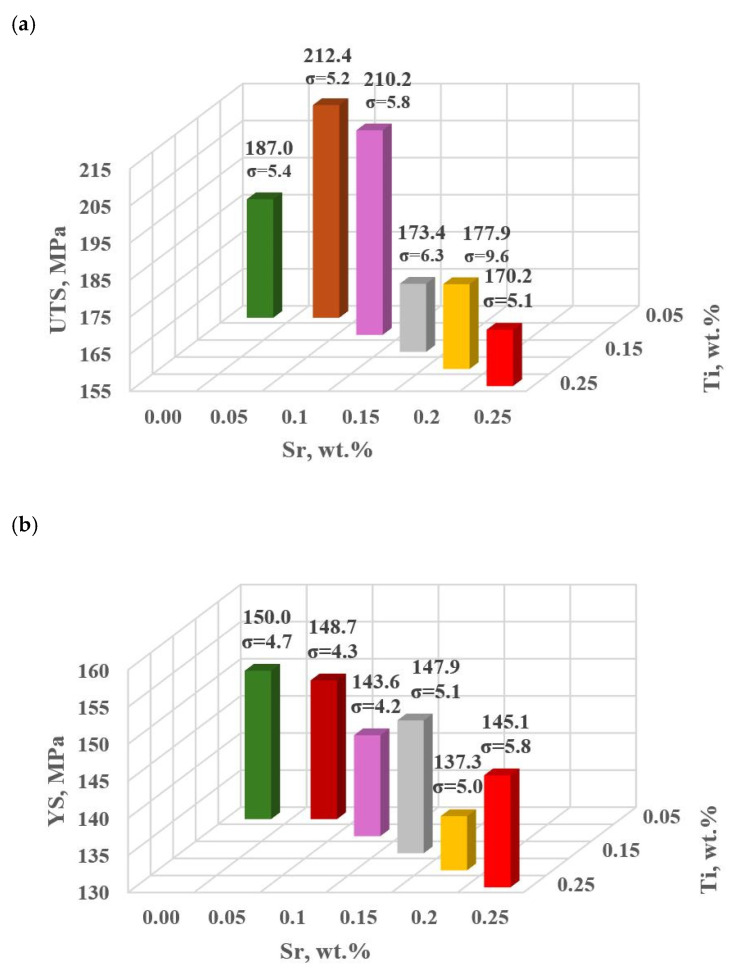

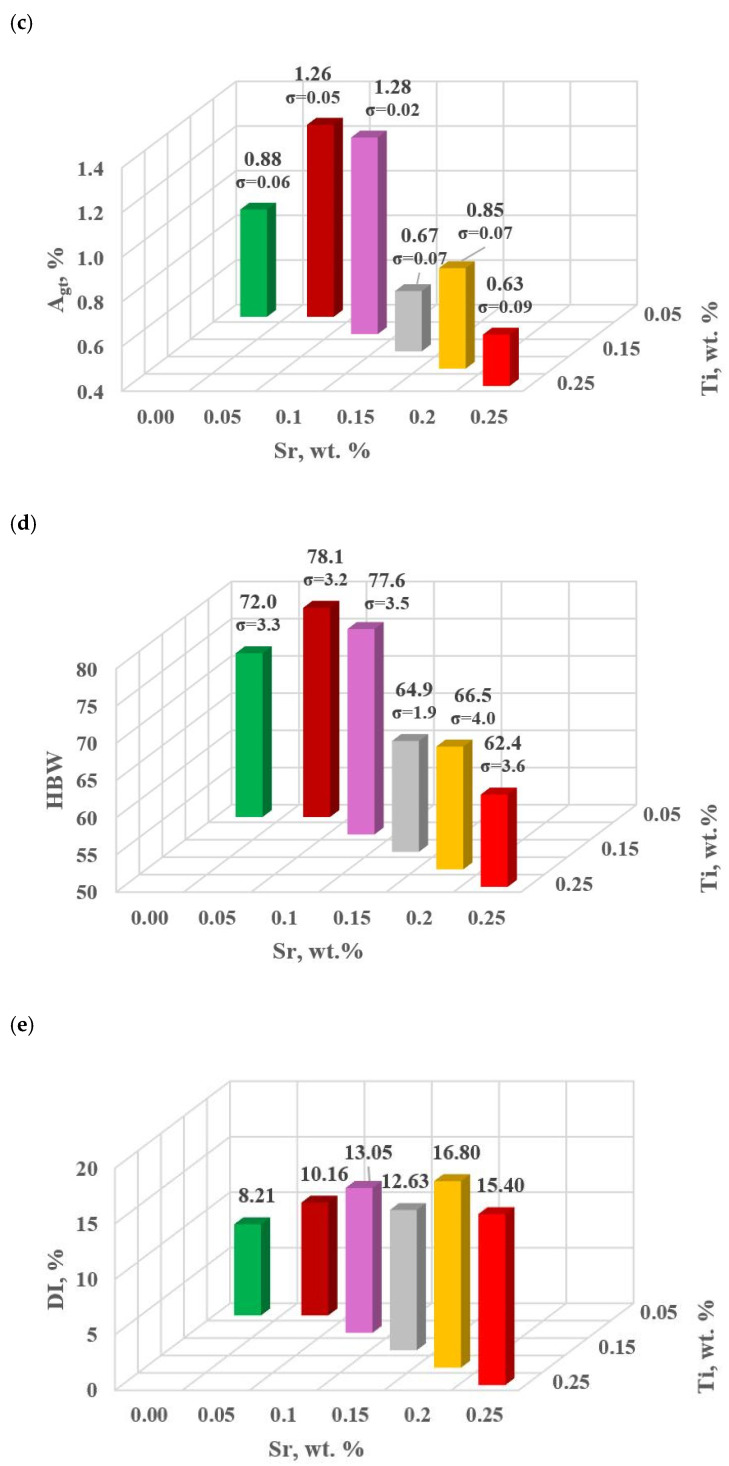
Basic mechanical properties of the tested EN AC-46000* alloy unmodified and modified according to the settings along the gradient vector: (**a**) tensile strength UTS; (**b**) yield strength YS; (**c**) relative elongation A_gt_; (**d**) hardness HBW; (**e**) density index DI.

Table 8 and Figure 12a–e show the basic mechanical properties and DI (density index) of the base alloy as well as with Sr and TiB.

Modifiers in amounts of 0.15–0.25 wt.% each (according to the experimental plan tasks: D0–D4) did not improve the properties of EN AC-46000* alloy. In general, the improvement of the tested mechanical properties was achieved after reducing Sr and Ti to 0.10 and 0.05 wt.%, according to the determined gradient of the largest changes in the object’s response to the Input quantities, (tasks D5 and D6, respectively). The maximum UTS = 212 MPa was observed for Sr and Ti at 0.05 wt.% each. For this modifier content, YS = 149 MPa value comparable to the base alloy was obtained, this was the highest value in the alloy with Sr and Ti. The relative elongation improved significantly in both D5 (0.10 wt.% Sr and Ti, each) and D6 (0.05 wt.% Sr and Ti, each) tasks. However, the maximum value (A_gt_ = 1.28%) was obtained in task D5. For Brinell hardness, both additional tasks D5 and D6 yielded similar values at 78 HB.

### 3.2. Determination of TDA Curves Parameters

**Figure 13:**
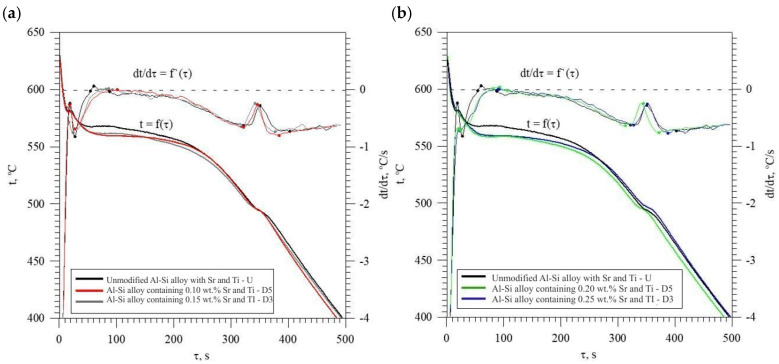
TDA curves of unmodified EN AC-46000* alloy and: (**a**) containing 0.10 (D5) and 0.15 (D3) % Sr and Ti each; (**b**) containing 0.20 (D0) and 0.25 (D1) % Sr and Ti each.

**Figure 14:**
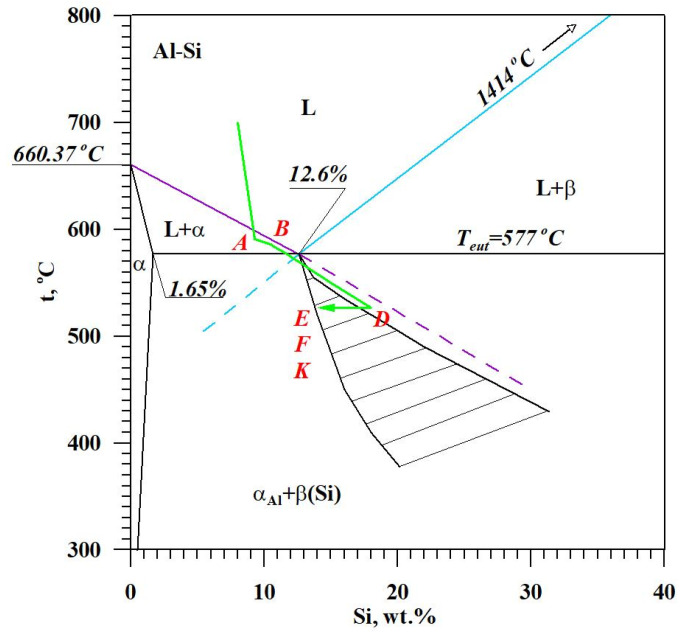
The crystallization scheme of hypoeutectic Al-Si alloy with Sr and TiB with the region of coupled growth of α_Al_ + β(Si) binary eutectic mixture.

Figure 13 shows TDA curves of the base alloy (see Figure 8) and alloys with Sr and Ti from 0.10 to 0.25 wt.% each. Their composition was made for equal maximum temperature of individual measurements t_max_ = const. From the data presented, there is a significant reduction in the crystallization temperature of α_Al_ + β(Si) binary eutectic mixture of alloys containing Sr and Ti additions relative to the base alloy (black color). The derivative curves primarily show a reduction in the thermal effect from the crystallization of the αAl primary phase of alloys containing 0.15–0.25 Sr and Ti each relative to the base alloy. An increase in the time of maximum thermal effect (point E) can also be observed for all alloys with Sr and Ti. The crystallization of hypoeutectic alloy with Sr and TiB, along with the transition to the coupled growth area of α_Al_ + β(Si) eutectic mixture is shown in Figure 14.

The coordinates of the characteristic points on TDA curves for all the alloys tested are shown in Table 9, Table 10, Table 11 and Table 12. The position of the characteristic points on these curves should be interpreted in the same way as for the base alloy (Figure 8). The analyzed coordinates of the characteristic points are the time of occurrence “τ”, temperature “t”, the first derivative “K = dt/dτ” of temperature “t” with respect to time “τ” and the second derivative “Z = d^2^t/dτ^2^” of temperature with respect to time. In runs D0-D4 (Table 9, Table 10, Table 11 and Table 12), the coordinates of the analyzed points on TDA from the experiment plan are presented (Table 1, gradient vector determination). Runs D5 and D6, highlighted in bold and italic font, because they show the coordinates of the analyzed points on TDA from the optimization stage of the studied alloy’s modification.

**Figure 15:**
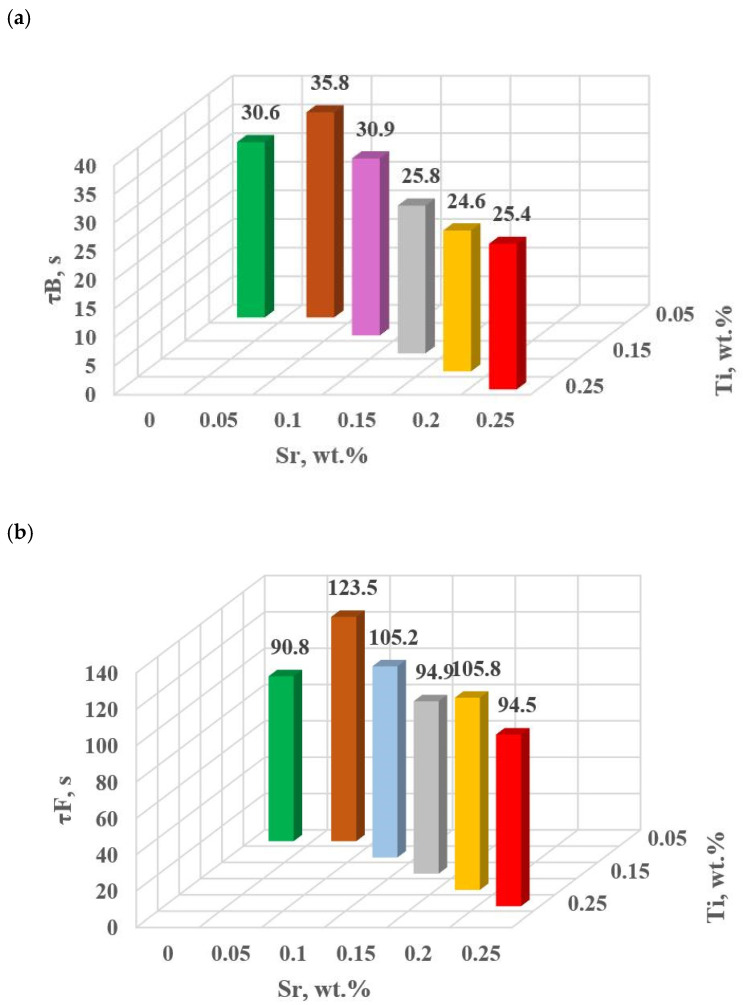

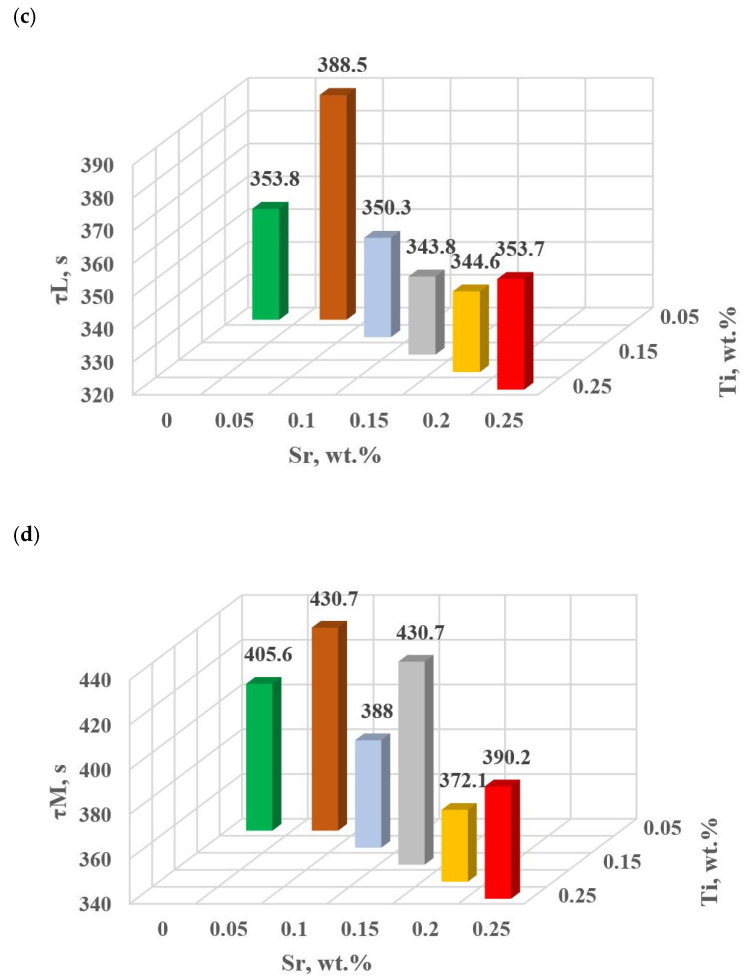
Time from the onset of TDA characteristic recording of hypoeutectic alloy with and without Sr and TiB: (**a**) τB, (**b**) τF, (**c**) τL, (**d**) τM.

**Figure 16:**
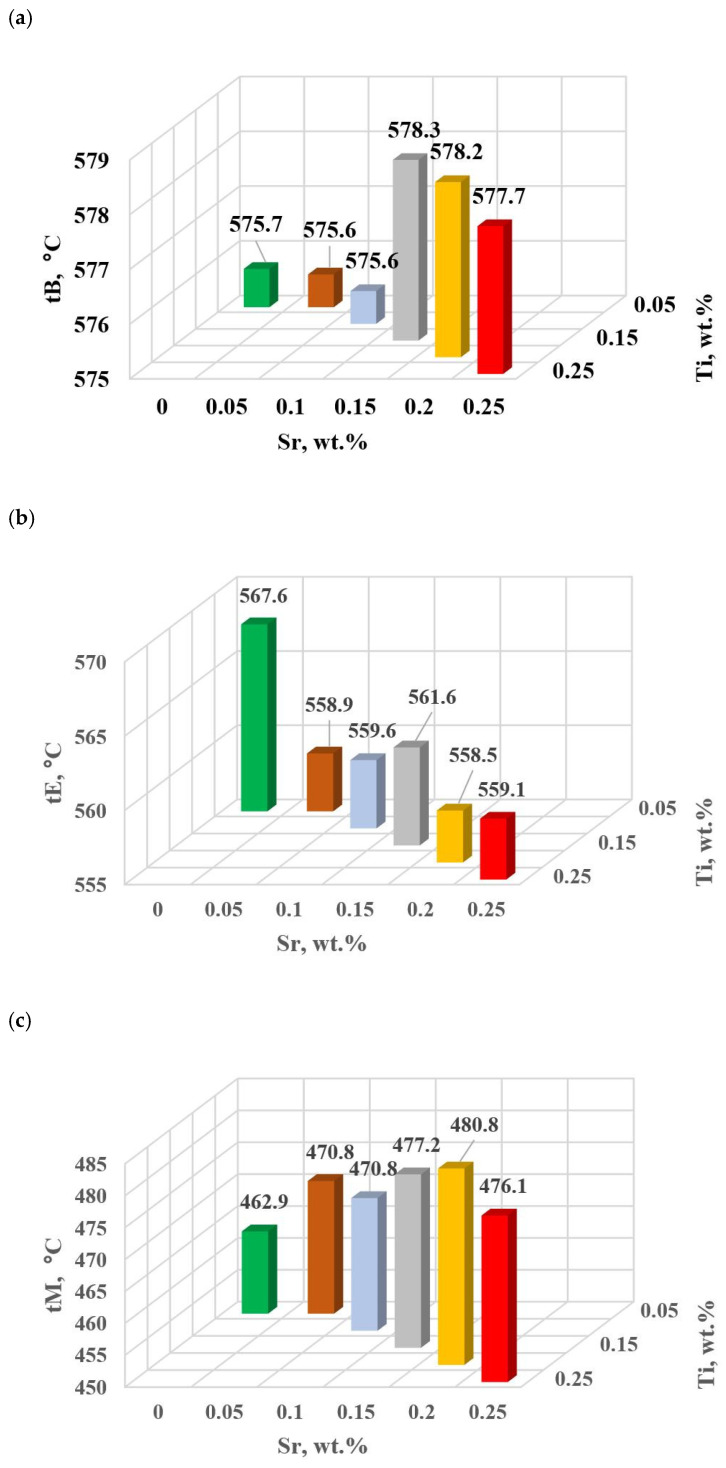
Temperature at characteristic points of hypoeutectic alloy without and with Sr and TiB: (**a**) tB, (**b**) tE, (**c**) tM.

**Figure 17:**
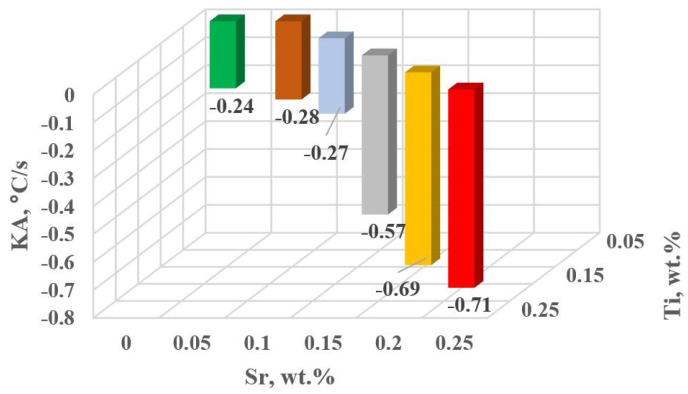
KA kinetics of thermal processes for the characteristic point A of hypoeutectic alloy without and with Sr and TiB.

**Figure 18:**
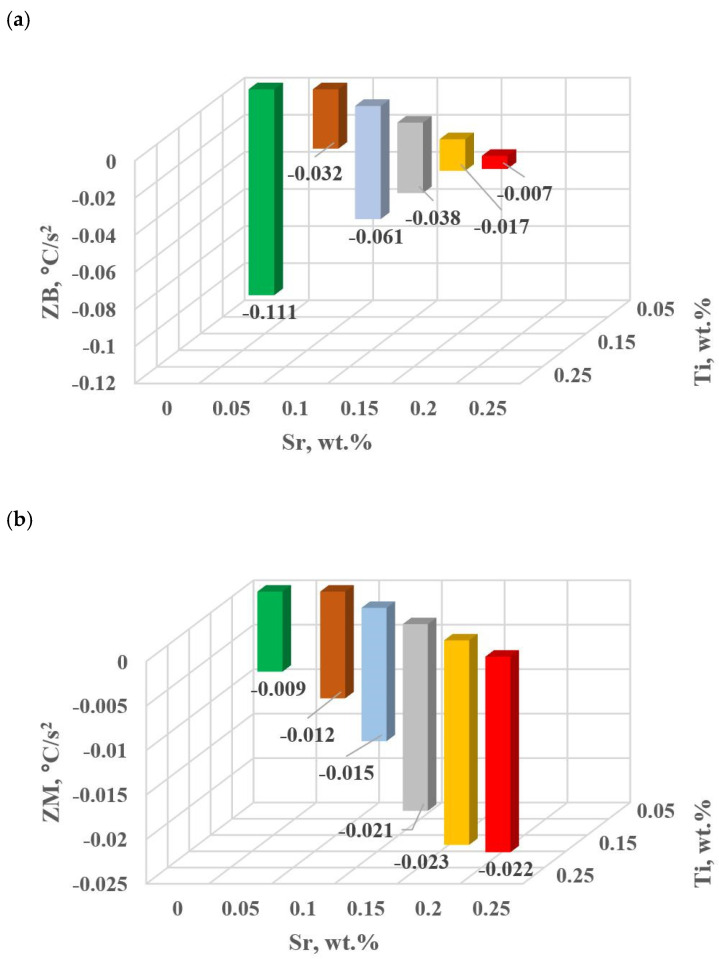
Dynamics of thermal processes for hypoeutectic alloy with and without Sr and TiB: (**a**) ZB crystallization of α_Al_ + β(Si) binary eutectic mixture, (**b**) ZM crystallization of complex eutectic mixture α_Al_ + β(Si) + Al_2_Cu.

The change in the values of the quantities determined on TDA characteristics entering the regression functions terms describing the variation of UTS, YS A, HBW and DI (Table 13) for tasks in the order of U, D6, D5, D3, D0 and D1 are shown in Figure 15a–d, Figure 16a–c, Figure 17 and Figure 18a,b, respectively, for:

- time values τB, τF, τL and τM (Figure 15a–d),
- temperature values tB, tE and tM (Figure 16a–c),
- kinetics values of thermal processes KA (Figure 17),
- dynamics values of thermal processes ZB and ZM (Figure 18a,b).

The study of Sr and TiB effect on the crystallization process of EN AC-46000* alloy (Table 2) shows that the crystallization of α_Al_ primary phase is sensitive to the presence of Sr and Ti in the alloy. In the range of primary crystallization (points A and B—Figure 8), decreasing Sr and Ti (as well as B) in the studied range 0.25–0.05 wt.% influenced a gradual increase in τB time (Figure 15a) describing the termination of α_Al_ phase crystallization. This is probably related to the fact that small amounts of modifiers decrease the dynamics of thermal processes ZB (Figure 18a) with increasing kinetics of thermal processes KA (Figure 17) and decrease the temperature tB (Figure 16a). At concentrations of about 0.05 wt.% Ti and Sr each, these changes result in high UTS (Figure 12a), A_gt_ (Figure 12c) and HBW (Figure 12d). Crystallization of α_Al_ primary phase also generates information regarding the change in the degree of the alloy gasification under study (DI (density index)). An increase in Ti, and especially Sr in the alloy under study, significantly increases its gasification, as evidenced by a significant increase in DI (density index) (Figure 12e). It should be emphasized that it is the increase in gasification (DI (density index)) that mainly influences the decrease in mechanical properties UTS, YS, A_gt_ and HBW, despite favorable changes in the microstructure of Sr and TiB-modified alloy, such as refinement of silicon precipitates.

In terms of α_Al_ + β(Si) binary eutectic crystallization (points B, D, E, F and K, Figure 8)—a significant correlation of time τF with elongation A_gt_ was identified (in addition to KA—primary crystallization). Point F describes the achievement of equilibrium in the thermal processes of binary eutectic (dt/dτ ≈ 0 °C/s) after the maximum of thermal effects (point E, dt/dτ = max °C/s). The increase in τF (Figure 15b) with the decrease in Sr and Ti concentration is influenced by the decrease in the dynamics of thermal processes ZB (Figure 18b) of the primary αAl phase crystallization, which delays the onset of nucleation and growth of α_Al_ + β(Si) binary eutectic mixture. It is significant for the analyzed alloy that for task U the temperatures describing the maximum thermal effect of eutectic mixture crystallization tD, tE (Figure 16b) and tF vary in the range 567–568 °C (Table 10), and with the presence of about 0.05% Sr and Ti (and more) each, they decrease to the range 558–562 °C. This shows that the modifiers force an increase in overcooling (6–9 °C) in the range of binary eutectic mixture crystallization. An increase in alloy gasification (DI index) is also partly responsible for the decrease in crystallization temperatures of this eutectic. The gas (H_2_) contained in the alloy reduces its thermal conductivity [25,26] which increases the time at the various stages of crystallization and cooling of the alloy (mixture: primary phase α_Al_ + nucleating eutectic mixture (α_Al_ + β(Si)) + liquid alloy + H_2_) in TDA probe.

In terms of the last stage of crystallization—the triple eutectic mixture α_Al_ + β(Si) + Al_2_Cu (points K, L and M—Figure 8)—it can be observed a strong increase in the dynamics of thermal processes ZM (Figure 18b) and an increase in the temperature tM (Figure 15c) as Sr and Ti concentration increases.

### 3.3. Microstructure Studies

**Figure 19:**
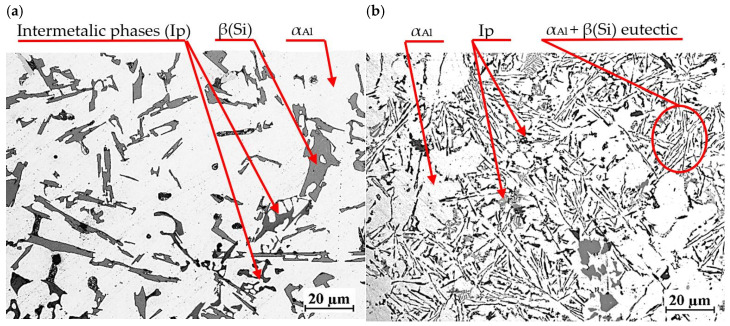
Microstructure of unmodified EN AC-46000* alloy: (**a**) from the shell mold (TDA probe); (**b**) from the permanent mold (static tensile test specimen).

Figure 19 shows the microstructure of EN AC-46000* base alloy from a shell mold (a) and a permanent mold (b).

Regardless of the casting technology used, the microstructure consists of α_Al_ solid solution dendrites, α_Al_ + β(Si) eutectic mixture as well as intermetallic phases. Eutectic silicon precipitations are characterized by lamellar morphology. The intermetallic phases Ip in the studied alloy are described in detail in [27,28,29,30,31]. These are mostly Cu- and Mg-rich phases Al_2_Cu, Mg_2_Si as well as Fe-rich phase Al_15_(Fe,Mn)_3_Si_2_. Higher cooling intensity in die casting leads to microstructure modification, including finer dendritic structure and more homogeneous distribution of the eutectic phase. This, in turn, improves the mechanical properties of the castings. Due to the different intensity of heat transfer from the casting in the two technologies considered, the microstructures shown in Figure 19a,b differ in the size of the phases. Less intense heat transfer through the shell mold resulted in a coarser-grained microstructure (Figure 19a) than in the case of the permanent mold casting (Figure 19b), where heat transfer was much more intense.

**Figure 20:**
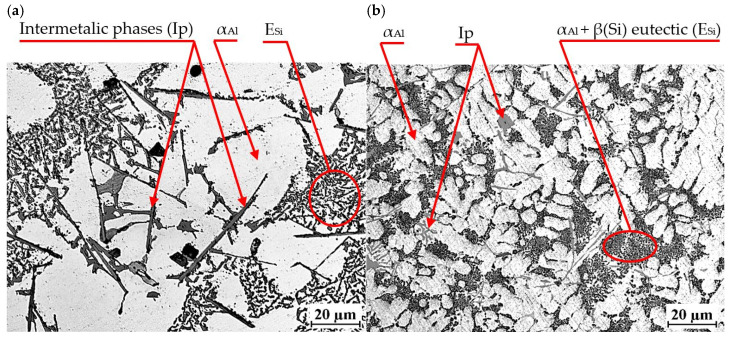
Microstructure of EN AC-46000* alloy with Sr and TiB: (**a**) from the shell mold (TDA probe); (**b**) from the permanent mold (static tensile test specimen).

An exemplary microstructure of EN AC-46000* alloy with Sr and TiB is shown in Figure 20a,b. This is the microstructure obtained in task D1, where Sr and Ti content was 0.25 wt.% each. In the other tasks carried out, a microstructure very similar to that shown in Figure 20 was obtained.

Again, the constituent phases are α_Al_ dendrites, α_Al_ + β(Si) eutectic mixture E_Si_ and Cu-, Mg- and Fe-rich intermetallic phases Al_2_Cu, Mg_2_Si and Al_15_(Fe,Mn)_3_Si_2_. After modification with strontium and titanium in amount of 0.25 wt.% each, the morphology of the eutectic silicon was significantly changed. Regardless of the casting technology used, Sr modification resulted in a change in silicon morphology from lamellar to so-called fibrous. On the metallographic section, β(Si) precipitates in the fibrous eutectic mixture can be seen as close to round or oval. Titanium, on the other hand, causes a reduction in the dimensions of α_Al_ phase dendrites. As shown in the paper, the modification of Al-Si alloy with Sr and TiB can significantly improve the microstructure and mechanical properties of high-pressure die castings. The Sr addition-induced change in the morphology of αAl + β(Si) eutectic mixture, leads to improved mechanical properties, such as tensile strength and impact strength. Strontium helps reduce pores and evenly distribute porosity in castings, which increases uniformity and surface quality. Modification with strontium improves alloy castability, which facilitates the casting process and reduces the risk of casting defects. The TiB addition-induced fragmentation and homogenization of the microstructure improves the tensile strength and hardness of the tested alloy. TiB helps eliminate hot cracks. In summary, modification of Al-Si alloys with Sr and TiB leads to a finer and more homogeneous microstructure, refinement of gas pores (without reducing H_2_ content in the alloy) as well as improved mechanical properties, making pressure castings more tough and of better quality [5,6,7,8,9,10,11,12,13,14].

**Figure 21:**
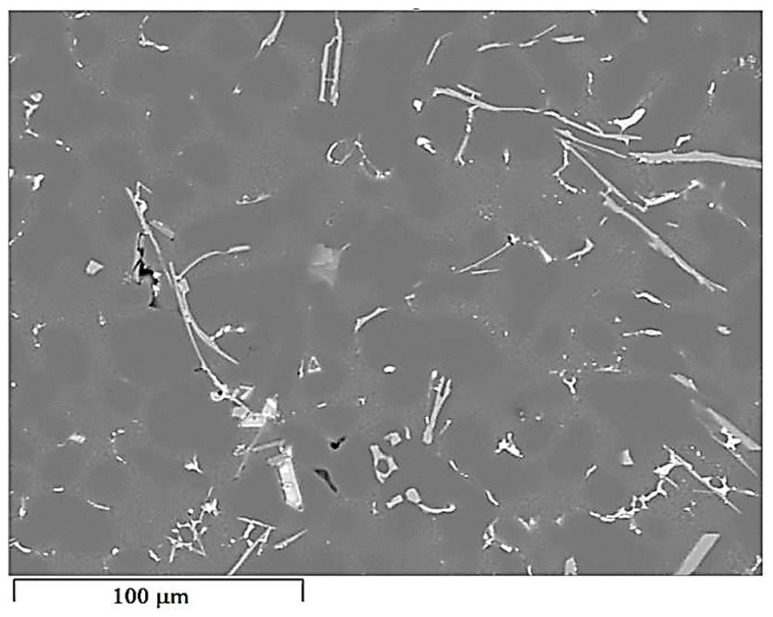
Area of EN AC-46000* alloy from permanent mold subjected to elements distribution test.

**Figure 22:**
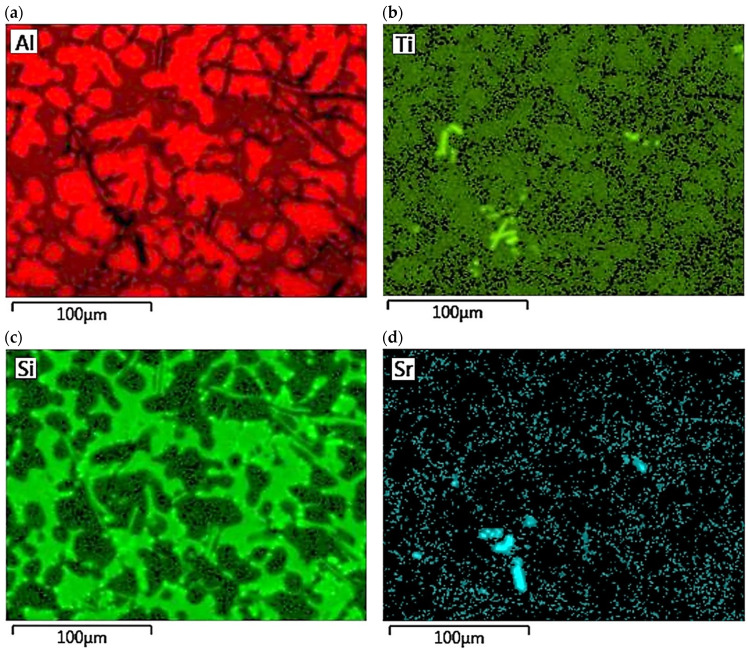

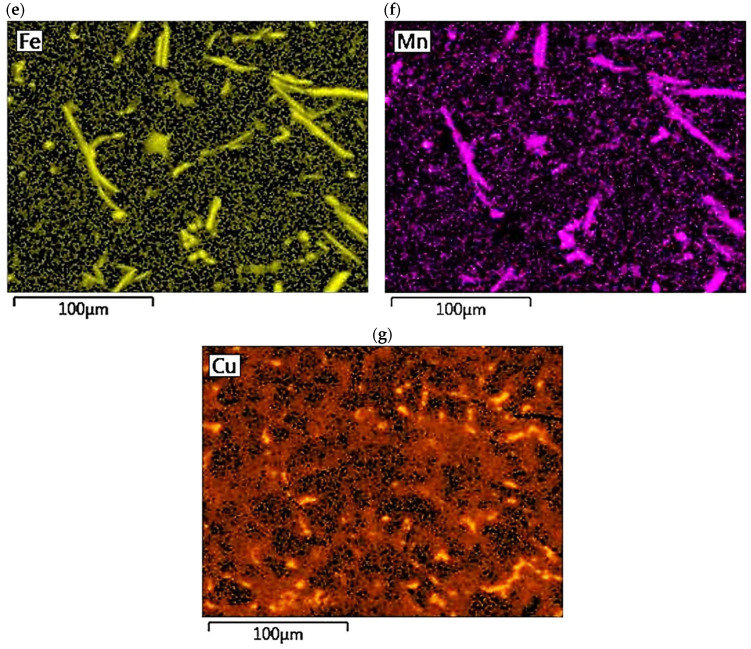
Distribution maps of analyzed elements in the studied alloy: (**a**) Al; (**b**) Ti; (**c**) Si; (**d**) Sr; (**e**) Fe; (**f**) Mn, (**g**) Cu.

The concentration distribution of Al, Si, Cu, Fe, Mn, as well as Sr and Ti was tested on a selected area of Al-Si alloy under study. Figure 21 shows the area of the microstructure studied, while Figure 22 maps the distribution of elements analyzed. From the data presented, there is a high concentration of Al in α_Al_ phase dendrites. There is also an elevated concentration of Ti. This is likely due to the formation of fine Al_3_Ti phase precipitation, which is widely considered to underlie heterogeneous nucleation of the α_Al_ phase [6,9,11,32]. Since boron was added into the alloy along with titanium in a 1/5 ratio, it is highly likely that the intermetallic phase TiB_2_ is also present in α_Al_ dendrites. This phase can also be a base for α_Al_ phase crystallization [6,9,11,32]. Elevated Si concentration, however, is found in α_Al_ + β(Si) eutectic mixture. Increased concentration of Sr can also be observed in these areas. Strontium added to modify the morphology of eutectic silicon into fine fibers shows increased content just in α_Al_ + β(Si) eutectic region. Presumably, Sr is involved in the formation of fine Al_2_Si_2_Sr phase particles in this area, which prevents harmful Si nucleation by neutralizing AlP particles. This phenomenon presumably alters Si growth kinetics and is often seen as a potential cause of the modifying Sr effect in Al-Si alloys [5,33,34]. Similarly, strontium can eliminate AlP particles by forming Sr_3_P_2_ phase [6,33,34,35]. High concentrations of Fe and Mn and Cu are assigned to areas of Fe_-rich_ and Cu_-rich_ intermetallic phases, respectively. Both Sr and Ti also occur in intermetallic phases of relatively large sizes. This is due to the high concentration of the afore-mentioned elements in the alloy. Such precipitations increase the brittleness of the alloy. In the case of strontium, these phases are Si_2_Sr and Al_2_Si_2_Sr. On the other hand, excessive amounts of titanium can cause the formation of Al_3_Ti phases of relatively large size.

### 3.4. Optimization of Modifiers Content

To determine the effect of Sr and Ti in the studied alloy on selected coordinates of points on TDA curves, stepwise multiple regression analysis was used. The identified Models-Equations, at the chosen significance level α, were significant and had significant coefficients in the Equations. The models were characterized by a high R^2^ coefficient and possibly low SEE standard deviation, and low MAE mean total error. This analysis allowed the development of mathematical models to determine the relationship between the coordinates of the characteristic points on the TDA curves and the Sr and Ti content. These models are shown in Table 13 and Table 14, respectively.

The developed regression function models (Table 13) make it possible, for the new melt, based on thermal and derivative analysis, to determine the values of mechanical (UTS, YS, A_gt_, HBW) and qualitative (DI) properties without testing on strength [36] and DI specimens. TDA also makes it possible to assess the correctness of modifications with Sr and TiB.

**Figure 23:**
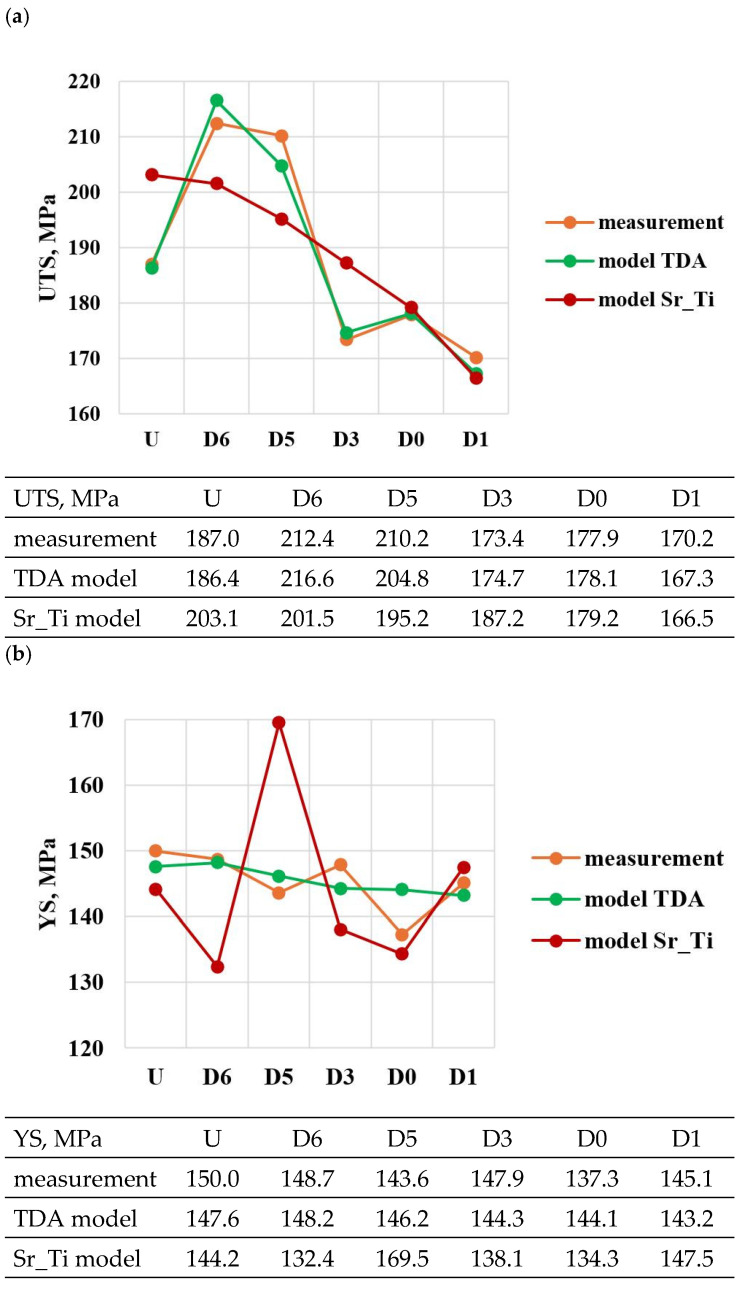

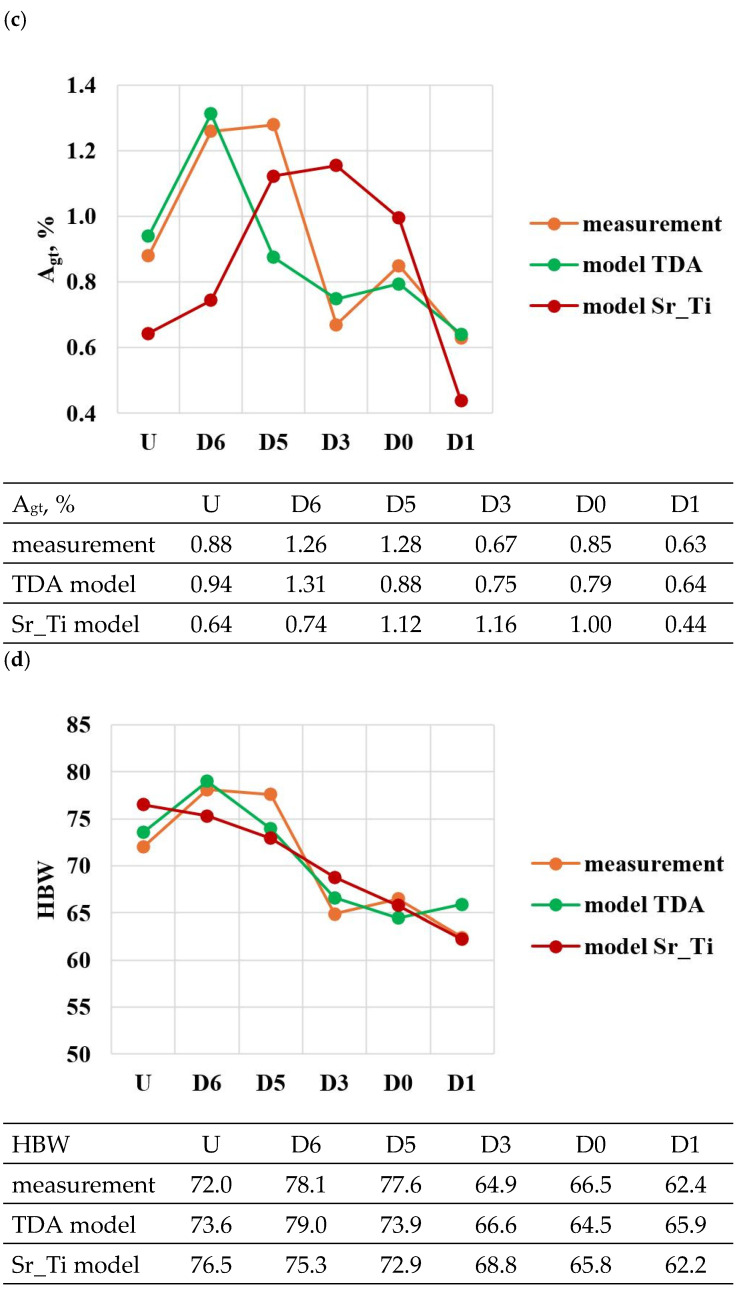

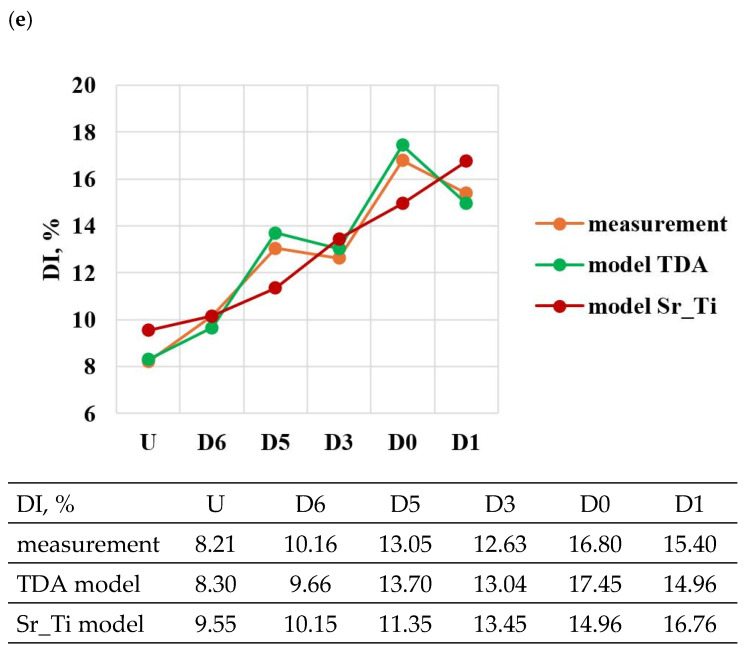
Summary of values from measurements (Table 4 and Table 8), TDA models (Table 13) and Sr_Ti models (Table 14) of the properties of hypoeutectic Al-Si alloy without and with Sr and TiB: (**a**) UTS, (**b**) YS, (**c**) A_gt_, (**d**) HBW, (**e**) DI.

A comparison of the solutions of the created models (TDA model—Table 13, Sr_Ti model—Table 14) with measured data for tasks in the order of U, D6, D5, D3, D0 and D1 is shown in Figure 23a–e. From the data presented, it can be seen that compared to the Sr_Ti models, the ATD models describe (lower SEE and MAE values) the variation of UTS, YS, A_gt_, HBW and DI properties much better indirectly (τX = f(Sr, Ti), tX = f(Sr, Ti), KX = f(Sr, Ti), ZX = f(Sr, Ti), where X selected by the TDA method a point on the dt/dτ characteristic) depending on changes in the amount of Sr and Ti.

According to the methodology, the developed Sr_Ti models were used to simulate changes in the properties of UTS, YS, A, HBW and DI (Table 15, Table 16, Table 17, Table 18 and Table 19) depending on the amount of Sr and Ti in the range of variation 0–0.1% increments in 0.01%. In the optimization, the amounts of Sr and Ti modifiers were considered for, respectively:

- UTS all tabulated values,
- YS values non-negative and greater than 100 MPa (according to EN 1706 [21] for AlSi9Cu alloy YS_min_ = 100 MPa) and only those where the condition YS < UTS is met for the same values of Sr and Ti,
- A_gt_ values of not less than 1% (according to EN 1706 for AlSi9Cu alloy A_min_ = 1%),
- HBW values not less than 75 (according to EN 1706 for AlSi9Cu alloy HBW_min_ = 75),
- DI all tabulated values.

After normalizing the stimulants UTS, YS, A_gt_, HBW and the destimulant DI according to relations (4) and (5), the value of the objective function Fo was determined according to formula (6). The maximum value of the objective function Fo in the Sr and Ti concentration system identified with the constraints described above is shown in Table 20. Table 20 also shows the values of the objective function in the immediate vicinity of the extreme value. The data presented shows that for Sr = 0.03 wt.% and Ti = 0.07 wt.% Fo_max_ = 2.58. For the identified optimal Sr and Ti concentration, the alloy properties are estimated as follows: UTS = 198 MPa, YS = 178 MPa, A_gt_ = 1%, HBW = 75 and DI = 10%.

Since it is difficult to keep the concentration of individual elements in the alloy “exactly on point”, the following range of variation in the concentration of Sr = 0.02–0.04 wt.% and Ti = 0.06–0.08 wt.% after modification in the EN AC-46000* alloy was proposed in order to achieve simultaneously the highest possible UTS, YS, A_gt_ and HBW properties and the lowest possible DI index value. In Table 15, Table 16, Table 17, Table 18 and Table 19, the selected ranges of Sr and Ti concentrations and the corresponding values of the analyzed properties or objective function in Table 20 are distinguished by bold+italic style.

Not over the entire range of variation in modifier concentrations of the developed mathematical models of individual properties generate acceptable values. Areas marked with different colors correspond to the particular physical sense criteria of the generated responses. For example, property values with a negative sign (areas in Table 16 marked in yellow) or with values less than the standard for a given alloy grade (areas in Table 16, Table 17 and Table 18 and in Table 20 marked in brown color) or, as in the case of YS, YS>UTS values (areas in Table 16 and Table 19 marked in violet).

In multi-criteria optimization, the aim is to find a solution that is a compromise between different criteria. Compromises must be made due to opposing criteria (e.g., UTS↑ to A_gt_↓), different mechanisms of action of modifiers (e.g., Sr modifies the eutectic mixture, while Ti grinds the grain), interactions between modifiers (too much of one modifier can interfere with another), or technological constraints (e.g., too high Sr concentration can lead to problems with alloy flowability or the formation of casting defects). Often there is no single solution that maximizes all properties at the same time, so it is necessary to find a balance point that provides acceptable results for all criteria.

The research conducted brings novelty by simultaneously using Sr and TiB as modifiers, which allows for a synergistic effect of improving microstructure and mechanical properties. Unlike previous studies that focused on single modifiers [6,7,12,29,30], our approach provides a better understanding of the interaction between Sr and TiB and their effects on the microstructure and mechanical properties of Al-Si alloys.

## 4. Summary

Modification of hypoeutectic Al-Si alloys with strontium (Sr) and titanium-boron (TiB) has a significant effect on the microstructure, mechanical properties and gas porosity. The advantages of complex modification of this type of alloy are:

- fragmentation of dendrites: the addition of TiB causes fragmentation of the α_Al_ phase, leading to a more homogeneous microstructure,
- change in eutectic morphology: strontium changes the morphology of the eutectic from lamellar to fibrous, which improves the mechanical properties,
- increase in strength: modification of Sr and TiB increases the tensile strength and hardness, improves ductility due to a more homogeneous and fibrous microstructure).

It is possible to obtain a lower DI (density index) because of TiB and Sr addition. However, complex modification carries several significant disadvantages, such as: the addition of Sr and TiB increases the cost of alloy production due to the high cost of these elements, the modification process requires precise control over the amount and manner of making additions, which can be difficult to achieve under production conditions. There are also potential problems with excess additives (over-modification): too many additives can lead to unfavorable changes in the microstructure, such as the formation of undesirable intermetallic phases, a renewed increase in porosity (an increase in DI (density index)).

In conclusion, modification of EN AC-46000 alloy with Sr and TiB brings many advantages in terms of microstructure and mechanical properties but is also associated with higher costs and greater complexity of the production process.

Thermal and derivative analysis of the alloy is sensitive to any changes in the technological process of making the liquid alloy—its physical and chemical state [33]. A change in chemical composition, carrying out treatments on the liquid alloy (e.g., modification, refining,...), or the lack thereof, are visible on TDA characteristics. Using the characteristic TDA quantities (t, τ, dt/dτ, d^2^t/dτ^2^), it is possible to develop statistical relationships—regression functions—that describe changes in the physical, or technological, properties of the alloy in the solid state. The developed models can be used to build a computer system for quality control of liquid alloy preparation. Immediately after measuring and recording the temperature of the alloy cooling and solidifying in the TDA probe, the preparation of the liquid alloy with the process guidelines can be carried out in a short time. As a result of non-compliance with the technological process, the pouring of the molds with the melt should be stopped and after pouring it from the transport ladle to the melting furnace, action should be taken to restore the correctness of the technological process. This avoids misguided melting and the associated cost of metal and molds that would yield castings with inappropriate properties.

Mathematical models describing the effect of Sr and/or Ti in the alloy developed based on TDA registrations (TDA model) are characterized by smaller SEE and MAE than models based solely on the actual content of these elements in the alloy (Sr_Ti model). This is mainly related to the fact that they consider chemical composition and liquid alloy treatments holistically. Nevertheless, the Sr_Ti models made it possible to carry out optimization of the amount of Sr and Ti after modification with AlSr10 and AlTi5B1 master alloys, as a function of simultaneous maximization of mechanical properties and minimization of the density index, using the lowest possible number of tasks in the experiment.

Validation of the recommended addition of Sr and Ti to EN AC-46000 alloy was carried out under production conditions. Strength specimens from the unmodified alloy and after optimal modification were made on a high-pressure die casting machine. The charge, consisting of ingots and returns (gating systems and flow-offs) in a 50%/50% ratio, was melted in a Striko Westofen MHW II N 2000/1500 shaft melting furnace. The alloy was degassed with Ecosal solid degasser followed by gas (N_2_) degassing. After the liquid melt was poured into the holding furnace of HPDC machine and a specimen was taken to test the density index, which amounted to DI = 0.87%. Then, strength specimens were made on HPDC machine. The alloy was then modified with about 0.06 wt.% Ti and about 0.04 wt.% Sr and degassed with a solid (Ecosal) and gas (N2) degasser. After the liquid melt was poured into the holding furnace of HPDC machine and a specimen was taken to test the density index, which amounted to DI = 0.95%. Again, strength specimens were made on HPDC machine. Table 21 shows the mechanical properties of EN AC-46000* alloy with optimal chemical composition, without and after optimal modification. The average values (Average) of the measurements of mechanical properties and their standard deviations (StdDev) are distinguished by font written in Bold+Italica style.

**Figure 24:**
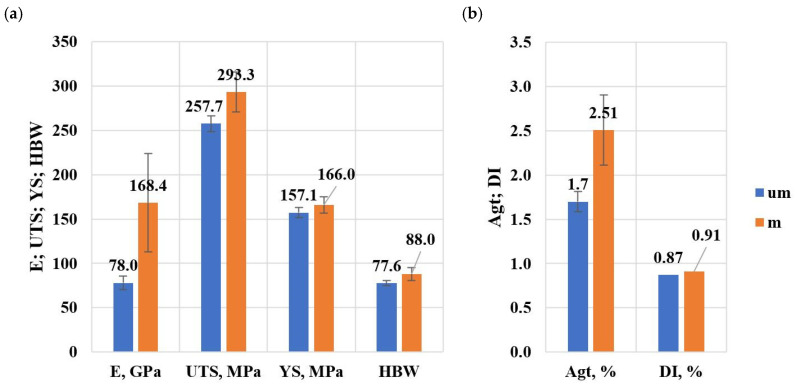
Summary of average values of mechanical properties and density index of the tested alloy: (**a**) E, UTS, YS, HBW, (**b**) A_gt_, DI.

Figure 24a,b shows a summary of the average values of mechanical properties and density index of the tested alloy.

There are opportunities for industrial application of an optimal modification process simultaneously with the addition of Sr and TiB to the alloy under study. The presented modification process is dedicated, first of all, in the process of casting high-quality castings for the following industries: automotive (e.g.,: engine blocks), aerospace (e.g.,: aircraft components, where high strength and low masses are required), electronics (e.g.,: housings and heat sinks), or construction (e.g.,: aluminum profiles, which require high strength and corrosion resistance).

The results of the research conducted have the potential to be widely applied in various industries, however, there are some limitations such as production costs, process complexity, and environmental impact. These should be considered when implementing these technologies on an industrial scale.

In conclusion, the research presented in the article makes a significant contribution to the development of Al-Si alloys HPDC technology, which is important for the automotive industry and other sectors requiring high-quality castings made using HPDC technology.

## 5. Conclusions

From the studies presented on the modification of EN AC-46000* alloy with Sr and TiB additives, the following conclusions are drawn:

- The modification process of Al-Si alloy with Sr and TiB was optimized as a function of simultaneous maximization of UTS, YS, A_gt_ and HBW and minimization of DI index, for Sr = 0.03 wt.% and Ti = 0.07 wt.%, the extreme values of properties in the die casting were estimated for UTS = 198 MPa, YS = 178 MPa, A_gt_ = 1%, HBW = 75 and DI = 10%, respectively,
- The optimal modifier concentration range was determined: Sr = 0.02–0.04 wt.% and Ti = 0.06–0.08 wt.%, with the proviso that to maintain a low DI index, the concentration of Sr = 0.02 wt.% in the melt should be kept as low as possible,
- To avoid excessive increase in Sr and Ti concentration after modification, the actual Sr and Ti concentration in the alloy should be strictly controlled when using process scrap of alloy modified with these additives,
- As expected, after optimal modification of EN AC-46000* alloy, higher mechanical properties were obtained on high-pressure die castings compared to those measured on die castings,
- On validation specimens (HPDC castings) from Sr and TiB modified alloy, average property values were obtained: E = 168.4 GPa (an increase relative to the unmodified alloy of about 116%), UTS = 293.3 MPa (an increase of about 14%), YS = 166 MPa (an increase of about 6%), A_gt_ = 2.51% (an increase of about 47%), HBW = 88 (an increase of about 13%) and DI = 0.91% (an increase of about 5%),
- Using the Box–Wilson method and multi-criteria optimization based on regression models, it was possible to optimize the modification process of EN AC-46000* alloy with Sr and TiB based on a small number of tasks in the experiment,
- Thermal and derivative analysis is an accurate tool for tracking the crystallization process after various liquid alloy treatments (e.g., modification) and can be used to build a liquid alloy quality control system.
- It should be noted that the presented results were obtained on specimens from gravity die with a compliant geometry, and carrying out effective modification of Sr and TiB of specific products obtained by HPDC technology will require verification of the results under production conditions.
- It is planned to adopt the developed technology of Sr and TiB modification for castings produced by HPDC technology characterized by certain geometric parameters, such as: overall dimensions, wall thickness and shape complexity.

## Figures and Tables

**Table 1:** Design of experiment of modification EN AC-46000* alloy.

Xs Factor	x_1_ = Ti, wt.%	x_2_ = Sr, wt.%
Task D0 (central level x_s_^0^)	0.20	0.20
Test step ∆x_s_	0.05	0.05
Upper level x_s_^0^ + ∆x_s_	0.25	0.25
Lower level x_s_^0^ − ∆x_s_	0.15	0.15
Task D1	0.25	0.25
Task D2	0.15	0.25
Task D3	0.15	0.15
Task D4	0.25	0.15

**Table 2:** Chemical composition of the EN AC-46000* basic alloy.

Chemical Composition, wt %											
Si	Cu	Zn	Fe	Mg	Mn	Ni	Cr	Ti	Sr	B	Al
9.46	2.17	0.86	0.85	0.29	0.20	0.074	0.026	0.041	<0.0001	0.0022	al.
–	–	–	–	–	–	–	–	–		–	
9.82	2.29	0.96	1.05	0.37	0.21	0.087	0.028	0.047		0.0024	

**Table 3:** Actual chemical composition of EN AC-46000 alloy obtained in individual melts.

Run	Chemical Composition, wt.%											
	Si	Fe	Cu	Mn	Mg	Cr	Ni	Zn	Ti	Sr	B	Al
U	9.55	0.85	2.24	0.207	0.34	0.027	0.074	0.09	0.04	<0.0001	0.0023	bal.
D0	9.46	1.03	2.25	0.201	0.32	0.027	0.082	0.87	0.19	0.18	0.0138	bal.
D1	9.51	0.92	2.26	0.199	0.34	0.026	0.086	0.96	0.27	0.24	0.0344	bal.
D2	9.55	1.01	2.19	0.204	0.34	0.027	0.083	0.88	0.15	0.25	0.0134	bal.
D3	9.54	1.04	2.25	0.203	0.33	0.027	0.083	0.89	0.14	0.13	0.0092	bal.
D4	9.53	0.85	2.17	0.201	0.32	0.026	0.079	0.86	0.28	0.15	0.0347	bal.
***D5***	***9.82***	***1.05***	***2.17***	***0.209***	***0.31***	***0.028***	***0.085***	***0.88***	***0.09***	***0.06***	***0.0051***	***bal.***
***D6***	***9.76***	***1.02***	***2.29***	***0.207***	***0.34***	***0.028***	***0.087***	***0.88***	***0.05***	***0.02***	***0.0029***	***bal.***

**Table 4:** Mechanical properties of the tested EN AC-46000* alloy and density index.

Melt/Task No.	UTS; MPa	YS; MPa	A_gt_; %	HBW	DI, %
U	187.0	150.0	0.88	72.0	8.21
D0	177.9	137.3	0.85	66.5	16.80
D1	170.2	145.1	0.63	62.4	15.40
D2	164.2	141.6	0.63	64.6	19.15
D3	173.4	147.9	0.67	64.9	12.63
D4	175.4	139.7	0.76	68.9	20.00

**Table 5:** Values of analyzed parameters of EN AC-46000 alloy* after standardization with the calculated value of the objective function for each task.

Melt/Task No.	nUTS	nYS	nA_gt_;	nHBW	nDI	Fo
D0	1.000	0.000	1.000	0.631	0.434	3.065
D1	0.438	0.736	0.000	0.000	0.624	1.798
D2	0.000	0.406	0.000	0.338	0.115	0.859
**D3**	**0.672**	**1.000**	**0.182**	**0.385**	**1.000**	**3.238**
D4	0.818	0.226	0.591	1.000	0.000	2.635

**Table 6:** Coordinates of gradient vectors for individual Outputs ($\vec{{\nabla Y}_{i}}i\nabla \vec{Y_{J}}$) and the resultant gradient ($\vec{\nabla GR}$) for the criterion of simultaneous maximization of stimulants (UTS, YS, A_gt_, HBW) and minimization of destimulant (DI).

Gradient Vector Plane Models	Gradient	Sr (axis x)	Ti (axis y)
UTS=185.85+19.2967·Ti−89.3124·Sr	∇UTS	[−89.312400;	19.296700]
YS=150.128−21.0887·Ti−17.4729·Sr	∇YS	[−17.472900;	−21.088700]
$A_{gt}=0.86474+0.179919\cdot Ti-1.01143\cdot Sr$	$\nabla A_{gt}$	[−1.011430;	0.179919]
HBW=71.3465+5.06702·Ti−35.9969·Sr	∇HBW	[−35.996900;	5.067020]
DI=7.91766+20.4796·Ti+23.9669·Sr	∇DI	[23.966900;	20.479600]
	∇GR	[−167.760530;	−17.024661]

**Table 7:** Theoretical setting values for tasks D5 and D6 according to Formula (6).

Melt No.	Ti, wt.%	Sr, wt.%
D5	0.19	0.10
D6	0.18	0.05

**Table 8:** Mechanical properties of the tested EN AC-46000* alloy and density index for unmodified alloy U and modified according to the settings for additional tasks D5 and D6.

Melt No.	UTS; MPa	YS; MPa	A_gt_; %	HBW	DI, %
U	187	150	0.88	72.0	8.21
D5	210.2	143.6	1.28	77.6	13.05
D6	212.4	148.7	1.26	78.1	10.16

**Table 9:** Crystallization time “τ” for individual characteristic points.

Melt/Run	τ, s							
	τA	τB	τD	τE	τF	τK	τL	τM
U	21.6	30.6	57.5	63.2	90.8	325.0	353.8	405.6
D0	20.7	24.6	80.9	93.0	105.8	313.2	344.6	372.1
D1	23.4	25.4	92.6	93.2	94.5	330.0	353.7	390.2
D2	19.0	25.4	70.2	82.4	97.8	285.9	310.9	344.2
D3	18.7	25.8	83.4	88.5	94.9	320.2	343.8	379.0
D4	20.8	25.2	75.8	84.8	102.0	288.3	318.4	345.2
***D5***	***22.0***	***30.9***	***105.2***	***105.2***	***105.2***	***326.0***	***350.3***	***388.0***
***D6***	***25.6***	***35.8***	***123.5***	***123.5***	***123.5***	***359.0***	***388.5***	***430.7***

**Table 10:** Temperature “t” at individual characteristic points.

Melt/Run	t, °C							
	tA	tB	tD	tE	tF	tK	tL	tM
U	581.6	575.7	567.2	567.6	568.2	508.6	493.8	462.9
D0	581.0	578.2	558.1	558.5	558.8	511.6	495.5	480.8
D1	579.2	577.7	559.1	559.1	559.3	507.5	496.7	476.1
D2	584.0	578.8	559.6	560.4	560.8	509.1	496.7	475.9
D3	583.4	578.3	561.5	561.6	561.8	507.7	496.7	477.2
D4	579.6	575.9	557.5	557.7	558.4	511.3	494.7	478.0
***D5***	***580.5***	***575.6***	***559.6***	***559.6***	***559.6***	***507.2***	***495.2***	***470.8***
***D6***	***577.4***	***572.9***	***558.9***	***558.9***	***558.9***	***509.4***	***495.6***	***470.8***

**Table 11:** Values of the first derivative “K = dt/dτ” of temperature “t” with respect to time “τ” at individual characteristic points.

Melt/Run	K, °C/s							
	KA	KB	KD	KE	KF	KK	KL	KM
U	−0.24	−0.82	−0.03	0.06	−0.03	−0.63	−0.28	−0.73
D0	−0.69	−0.73	0.01	0.04	−0.02	−0.64	−0.25	−0.76
D1	−0.71	−0.73	0.00	0.00	0.00	−0.63	−0.26	−0.77
D2	−0.57	−0.81	−0.01	0.06	0.00	−0.67	−0.33	−0.79
D3	−0.57	−0.75	0.01	0.02	0.00	−0.65	−0.24	−0.74
D4	−0.81	−0.83	−0.02	0.04	0.00	−0.68	−0.29	−0.82
***D5***	***−0.27***	***−0.69***	***0.00***	***0.00***	***0.00***	***−0.66***	***−0.26***	***−0.81***
***D6***	***−0.28***	***−0.50***	***0.00***	***0.00***	***0.00***	***−0.61***	***−0.26***	***−0.75***

**Table 12:** Second derivative “Z = d^2^t/dτ^2^” of temperature “t” with respect to time “τ” at individual characteristic points.

Melt/Run	Z, °C/s^2^							
	ZA	ZB	ZD	ZE	ZF	ZK	ZL	ZM
U	0.211	−0.111	0.027	-	−0.001	-	0.031	−0.009
D0	0.203	−0.017	0.022	-	−0.002	-	0.028	−0.023
D1	0.102	−0.007	0.016	-	−0.002	-	0.020	−0.022
D2	0.279	−0.053	0.021	-	−0.004	-	0.016	−0.022
D3	0.162	−0.038	0.015	-	−0.003	-	0.023	−0.021
D4	0.213	−0.009	0.018	-	−0.002	-	0.026	−0.032
***D5***	***0.266***	***−0.061***	***0.015***	***-***	***−0.006***	***-***	***0.022***	***−0.015***
***D6***	***0.172***	***−0.032***	***0.009***	***-***	***−0.003***	***-***	***0.022***	***−0.012***

**Table 13:** Regression Equations for mechanical properties UTS, YS, A_gt_, HB and qualitative DI and their statistical measures (for α = 0.05 and υ_2_ = 8) as a function of characteristic values (t, K, τ, Z) identified on TDA curves.

Property	Equation—TDA Model	υ_1_	F(α, υ_1_, υ_2_)	F_model_	a_i_	P__value ai_	R^2^	SEE	MAE
UTS, MPa	UTS=2557.46+71.452·KA+1.69547·τL−1.48349·τM−4.08562·tB	3	2.92 *	22.01	a_0_	0.0538	96.71	5.04	2.48
					a_1_	0.0238			
					a_2_	0.0381			
					a_3_	0.0425			
					a_4_	0.0624			
YS, MPa	YS=0.332046·tM+676.109·ZM	6	3.58	5883.92	a_1_	0.0000	99.95	3.76	2.74
					a_2_	0.0151			
A_gt_, %	$A_{gt}=0.733933\cdot KA+0.0122952\cdot \tau F$	6	3.58	231.39	a_1_	0.0072	98.72	0.12	0.09
					a_2_	0.0000			
HBW	$HBW=110.976-\frac{1144.42}{\tau B}$	6	3.58	24.62	a_1_	0.0000	80.40	2.86	2.26
					a_2_	0.0025			
DI, %	DI=988.996−24.2474·KA −1.77532·tE−190.579·ZB	4	3.84	57.03	a_0_	0.0021	97.72	0.83	0.51
					a_1_	0.0006			
					a_2_	0.0021			
					a_3_	0.0032			

* α = 0.10.

**Table 14:** Regression Equations for mechanical properties UTS, YS, A_gt_, HB and qualitative DI and their statistical measures (for α = 0.05 and υ_2_ = 6) as a function of Ti and/or Sr mass concentration in alloy.

Property	Equation—Sr_Ti model	υ_1_	F(α, υ_1_, υ_2_)	F_model_	a_i_	P__value ai_	R^2^	SEE	MAE
UTS, MPa	UTS=209.513−159.465·Ti	4	3.18 *	5.03	a_0_	0.0000	55.72	13.80	9.88
					a_1_	0.0883			
YS, MPa	YS=3603.83·Ti−2150.32·Sr −4775.49·Ti·Sr	3	4.76	109.29	a_1_	0.0019	99.15	19.00	10.54
					a_2_	0.0202			
					a_3_	0.0184			
A_gt_, %	$A_{gt}=16.0984\cdot Ti-60.3219\cdot Ti\cdot Sr$	4	4.53	16.86	a_1_	0.0080	89.39	0.38	0.26
					a_3_	0.0209			
HBW	HBW=76,5182−59,6026·Sr	4	4.53	9.77	a_0_	0.0000	70.95	4.02	2.78
					a_2_	0.0353			
DI, %	DI=9.55067+30.0253·Sr	4	4.53	15.03	a_0_	0.0008	78.98	1.63	1.18
					a_2_	0.0179			

* α = 0.10.

**Table 15:** Prediction of UTS changes (according to the regression model in Table 14) vs. Ti and Sr concentration.

	Sr	0.00	0.01	***0.02***	***0.03***	***0.04***	0.05	0.06	0.07	0.08	0.09	0.10
Ti												
0.00		210	210	210	210	210	210	210	210	210	210	210
0.01		208	208	208	208	208	208	208	208	208	208	208
0.02		206	206	206	206	206	206	206	206	206	206	206
0.03		205	205	205	205	205	205	205	205	205	205	205
0.04		203	203	203	203	203	203	203	203	203	203	203
0.05		202	202	202	202	202	202	202	202	202	202	202
***0.06***		200	200	***200***	***200***	***200***	200	200	200	200	200	200
***0.07***		198	198	***198***	**198**	***198***	198	198	198	198	198	198
***0.08***		197	197	***197***	***197***	***197***	197	197	197	197	197	197
0.09		195	195	195	195	195	195	195	195	195	195	195
0.10		194	194	194	194	194	194	194	194	194	194	194

**Table 16:** Prediction of YS changes (according to the regression model in Table 14) vs. Ti and Sr concentration.

	Sr	0.00	0.01	***0.02***	***0.03***	***0.04***	0.05	0.06	0.07	0.08	0.09	0.10
Ti												
0.00		0	−22	−43	−65	−86	−108	−129	−151	−172	−194	−215
0.01		36	14	−8	−30	−52	−74	−96	−118	−140	−162	−184
0.02		72	50	27	5	−18	−40	−63	−85	−108	−130	−153
0.03		108	85	62	39	16	−7	−30	−52	−75	−98	−121
0.04		144	121	97	74	50	27	4	−20	−43	−67	−90
0.05		180	156	132	109	85	61	37	13	−11	−35	−59
***0.06***		216	192	***167***	***143***	***119***	94	70	46	21	−3	−27
***0.07***		252	227	***203***	**178**	***153***	128	103	78	53	29	4
***0.08***		288	263	***238***	***212***	***187***	162	136	111	86	60	35
0.09		324	299	273	247	221	195	170	144	118	92	66
0.10		360	334	308	282	255	229	203	176	150	124	98

**Table 17:** Prediction of A_gt_ changes (according to the regression model in Table 14) vs. Ti and Sr concentration.

	Sr	0.00	0.01	***0.02***	***0.03***	***0.04***	0.05	0.06	0.07	0.08	0.09	0.10
Ti												
0.00		0.0	0.0	0.0	0.0	0.0	0.0	0.0	0.0	0.0	0.0	0.0
0.01		0.2	0.2	0.1	0.1	0.1	0.1	0.1	0.1	0.1	0.1	0.1
0.02		0.3	0.3	0.3	0.3	0.3	0.3	0.2	0.2	0.2	0.2	0.2
0.03		0.5	0.5	0.4	0.4	0.4	0.4	0.4	0.4	0.3	0.3	0.3
0.04		0.6	0.6	0.6	0.6	0.5	0.5	0.5	0.5	0.5	0.4	0.4
0.05		0.8	0.8	0.7	0.7	0.7	0.7	0.6	0.6	0.6	0.5	0.5
***0.06***		1.0	0.9	***0.9***	***0.9***	***0.8***	0.8	0.7	0.7	0.7	0.6	0.6
***0.07***		1.1	1.1	***1.0***	**1.0**	***1.0***	0.9	0.9	0.8	0.8	0.7	0.7
***0.08***		1.3	1.2	***1.2***	***1.1***	***1.1***	1.0	1.0	1.0	0.9	0.9	0.8
0.09		1.4	1.4	1.3	1.3	1.2	1.2	1.1	1.1	1.0	1.0	0.9
0.10		1.6	1.5	1.5	1.4	1.4	1.3	1.2	1.2	1.1	1.1	1.0

**Table 18:** Prediction of HBW changes (according to the regression model in Table 14) vs. Ti and Sr concentration.

	Sr	0	0.01	***0.02***	***0.03***	***0.04***	0.05	0.06	0.07	0.08	0.09	0.10
Ti												
0		77	76	75	75	74	74	73	72	72	71	71
0.01		77	76	75	75	74	74	73	72	72	71	71
0.02		77	76	75	75	74	74	73	72	72	71	71
0.03		77	76	75	75	74	74	73	72	72	71	71
0.04		77	76	75	75	74	74	73	72	72	71	71
0.05		77	76	75	75	74	74	73	72	72	71	71
***0.06***		77	76	***75***	***75***	***74***	74	73	72	72	71	71
***0.07***		77	76	***75***	***75***	***74***	74	73	72	72	71	71
***0.08***		77	76	***75***	***75***	***74***	74	73	72	72	71	71
0.09		77	76	75	75	74	74	73	72	72	71	71
0.10		77	76	75	75	74	74	73	72	72	71	71

**Table 19:** Prediction of DI changes (according to the regression model in Table 14) vs. Ti and Sr concentration.

	Sr	0	0.01	***0.02***	***0.03***	***0.04***	0.05	0.06	0.07	0.08	0.09	0.10
Ti												
0		10	10	10	10	11	11	11	12	12	12	13
0.01		10	10	10	10	11	11	11	12	12	12	13
0.02		10	10	10	10	11	11	11	12	12	12	13
0.03		10	10	10	10	11	11	11	12	12	12	13
0.04		10	10	10	10	11	11	11	12	12	12	13
0.05		10	10	10	10	11	11	11	12	12	12	13
***0.06***		10	10	***10***	***10***	***11***	11	11	12	12	12	13
***0.07***		10	10	***10***	***10***	***11***	11	11	12	12	12	13
***0.08***		10	10	***10***	***10***	***11***	11	11	12	12	12	13
0.09		10	10	10	10	11	11	11	12	12	12	13
0.10		10	10	10	10	11	11	11	12	12	12	13

**Table 20:** Fo value (Formula (9)) vs. Ti and Sr concentration.

	Sr	0	0.01	***0.02***	***0.03***	***0.04***	0.05	0.06	0.07	0.08	0.09	0.10
Ti												
0												
0.01												
0.02												
0.03												
0.04												
0.05												
***0.06***				***2.61***	***2.09***	***1.57***						
***0.07***				***3.12***	**2.58**	***2.05***						
***0.08***				***3.62***	***3.08***	***2.53***						
0.09												
0.10												

**Table 21:** Mechanical properties of the tested alloy without and after optimal modification (condition: um—unmodified alloy, m—modified alloy).

No. Probe	State	E, GPa	UTS, MPa	YS, MPa	A_gt_, %	HBW
1	um	71.5	248.5	151.7	1.63	75.2
2		86.5	258.6	163.0	1.64	77.1
3		76.1	265.9	156.6	1.83	80.6
	***Average***	***78.0***	***257.7***	***157.1***	***1.70***	***77.6***
	***StdDev***	***7.68***	***8.74***	***5.65***	***0.11***	***2.74***
1	m	188.5	289.9	167.9	2.05	87.3
2		211.2	272.5	156.1	2.71	81.1
3		105.6	317.6	174.1	2.76	95.6
	***Average***	***168.4***	***293.3***	***166.0***	***2.51***	***88.0***
	***StdDev***	***55.59***	***22.75***	***9.14***	***0.40***	***7.28***

## Data Availability

The original contributions presented in this study are included in the article. Further inquiries can be directed to the corresponding authors.
